# A Recurrent Mutation in Anaplastic Lymphoma Kinase with Distinct Neoepitope Conformations

**DOI:** 10.3389/fimmu.2018.00099

**Published:** 2018-01-30

**Authors:** Jugmohit S. Toor, Arjun A. Rao, Andrew C. McShan, Mark Yarmarkovich, Santrupti Nerli, Karissa Yamaguchi, Ada A. Madejska, Son Nguyen, Sarvind Tripathi, John M. Maris, Sofie R. Salama, David Haussler, Nikolaos G. Sgourakis

**Affiliations:** ^1^Department of Chemistry and Biochemistry, University of California, Santa Cruz, Santa Cruz, CA, United States; ^2^Department of Biomolecular Engineering, University of California, Santa Cruz, Santa Cruz, CA, United States; ^3^Division of Oncology, Center for Childhood Cancer Research, Children’s Hospital of Philadelphia, Philadelphia, PA, United States; ^4^Department of Computer Science, University of California, Santa Cruz, Santa Cruz, CA, United States; ^5^Department of Molecular, Cell, and Developmental Biology, University of California, Santa Cruz, Santa Cruz, CA, United States; ^6^Department of Microbiology, Perelman School of Medicine, University of Pennsylvania, Philadelphia, PA, United States; ^7^Howard Hughes Medical Institute, University of California, Santa Cruz, Santa Cruz, CA, United States

**Keywords:** neoepitopes, MHC class I, human leukocyte antigens, structural biology, computational biology, cancer, T cell receptor

## Abstract

The identification of recurrent human leukocyte antigen (HLA) neoepitopes driving T cell responses against tumors poses a significant bottleneck in the development of approaches for precision cancer therapeutics. Here, we employ a bioinformatics method, Prediction of T Cell Epitopes for Cancer Therapy, to analyze sequencing data from neuroblastoma patients and identify a recurrent anaplastic lymphoma kinase mutation (*ALK* R1275Q) that leads to two high affinity neoepitopes when expressed in complex with common HLA alleles. Analysis of the X-ray structures of the two peptides bound to HLA-B*15:01 reveals drastically different conformations with measurable changes in the stability of the protein complexes, while the self-epitope is excluded from binding due to steric hindrance in the MHC groove. To evaluate the range of HLA alleles that could display the *ALK* neoepitopes, we used structure-based *Rosetta* comparative modeling calculations, which accurately predict several additional high affinity interactions and compare our results with commonly used prediction tools. Subsequent determination of the X-ray structure of an HLA-A*01:01 bound neoepitope validates atomic features seen in our *Rosetta* models with respect to key residues relevant for MHC stability and T cell receptor recognition. Finally, MHC tetramer staining of peripheral blood mononuclear cells from HLA-matched donors shows that the two neoepitopes are recognized by CD8+ T cells. This work provides a rational approach toward high-throughput identification and further optimization of putative neoantigen/HLA targets with desired recognition features for cancer immunotherapy.

## Introduction

Cancer immunotherapy harnesses a patient’s CD4+ and CD8+ T cell responses toward peptide neoantigens, which are displayed on the surface of tumor cells by major histocompatibility complex molecules [MHC, termed human leukocyte antigen (HLA) in humans] (1). In the endogenous presentation pathway (MHC class I), abundantly expressed intracellular proteins are processed by the immunoproteasome and proteasome to yield short peptide fragments that are transported into the endoplasmic reticulum and assembled together with the MHC-I heavy chain and β2-microglobulin light chain (β_2_m) by the peptide-loading complex (2). The resulting peptide/MHC complexes (p/MHC) are further trafficked through the Golgi and eventually displayed on the cell surface, where they are surveilled by CD8+ cytotoxic T cells (CTLs) through specific interactions with αβ T cell receptors (TCRs) (3). Through this process, a large and heterogeneous pool of p/MHC antigens is continuously generated in healthy, pathogen infected, or tumor cells as a means of displaying a cell’s peptide repertoire to the immune system (4). The display of high affinity peptides expressed exclusively by the tumor (i.e., neoepitopes) on MHC molecules can elicit specific CTL responses, which forms the basis of several established immunotherapies against cancers (5, 6). One such therapy utilizes *in vitro*-activated, autologous CTLs to selectively target tumor cells (7). Alternatively, vaccines can be designed based on known antigens or CTLs can be engineered to introduce TCRs with desired specificities toward displayed tumor antigens (8). In all cases, neoepitopes derived from commonly mutated oncogenic proteins are well-suited immunotherapy targets if they have high affinity interactions with MHC alleles that are prevalent in the population (9).

Neuroblastoma (NBL) is a widely metastatic form of cancer that affects the development of nerve cells that comprise the sympathetic nervous system, primarily in patients younger than 10 years old (10). High-risk NBL has a survival rate of less than 50% after intensive chemotherapy, radiation therapy, and other approved treatments (11). In addition, patients responding positively to radiation treatments generally do not achieve long-term survival and suffer from cancer relapse, often with an increased rate of tumor mutations (12). Sequencing studies focusing on NBL of all stages indicate a wide spectrum of somatic mutations in tumors, which poses a significant challenge for the development of targeted therapeutics (13). Notably, mutations in the anaplastic lymphoma kinase gene (*ALK)* have been implicated in 9.2% of 240 NBL cases with available whole exome, genome, and transcriptome sequencing data from the TARGET (Therapeutically Applicable Research to Generate Effective Treatments) initiative (12). This and other sequencing data support *ALK* as the target with the highest mutation rate among high-risk NBL patients (10, 12, 14). Furthermore, genome sequencing of relapsed NBL tumors demonstrates retention of ALK mutations and/or acquisition of an *ALK* mutation in 14/54 (15) and 10/23 (16) samples. Such *ALK* mutations have been shown to hyperactivate the RAS–MAPK signaling pathway in NBL, driving cancer formation (17). More recent studies have also shown evidence of ALK overexpression in NBL tumors making it a viable target for CAR-mediated immunotherapy along with other targeted T cell therapies (18). Immunotherapy offers an attractive approach toward NBL treatment. However, despite significant progress in identifying recurrent mutations toward understanding the genetic basis of NBL, important molecular details regarding derived neoantigen/HLA interactions remain unknown, which further limits the development of targeted T cell therapies (11).

Here, we use our recently developed multilayered bioinformatics pipeline, Prediction of T Cell Epitopes for Cancer Therapy (ProTECT), to predict therapeutically relevant antigens in NBL tumors. ProTECT analysis of 106 patient samples from the NBL TARGET cohort identifies a recurring “hotspot” mutation in the *ALK* protein (R1275Q), together with its specificity toward common HLA alleles. Specifically, two putative peptide sequences with the R1275Q mutation, a nonamer and a decamer, are predicted to bind HLA-B*15:01 with high affinity according to consensus methods (19, 20). X-ray structures of the two neoepitopes in complex with HLA-B*15:01 reveal a drastic change in peptide conformation, which correlates with increased thermal stability of the decamer neoepitope/HLA complex. For the self-peptide, unfavorable interactions between the peptide and residues in the MHC-binding groove prevent the formation of a stable complex. To evaluate the potential of the two ALK neoepitopes to interact with additional HLA alleles and predict structural features relevant for recognition by TCRs, we develop a high-throughput comparative modeling approach using the program *Rosetta*. Independent crystallographic analysis of a decamer-bound HLA-A*01:01 complex reveals a peptide conformation, which falls extremely close to our *Rosetta* model (within 1.1 Å backbone RMSD). Finally, tetramer staining of peripheral blood mononuclear cells (PBMCs) from HLA-B*15:01-matched donors followed by flow cytometry analysis shows that the two different neoantigen conformations are recognized by CD8+ T cells. Taken together, our bioinformatics analysis, *in vitro* and structural characterization, computational modeling, and T cell recognition analysis illustrate a powerful approach toward high-throughput identification and optimization of broadly displayed putative neoantigen/HLA targets for further development toward cancer immunotherapy. Results from this approach provide strong evidence for broad HLA display of recurrent *ALK-*derived neoantigens expressed in NBL tumors and further suggest that the presentation of distinct neoepitope conformations in the HLA groove could drive specific CD8+ T cell responses in patients.

## Results

### Identification of *ALK* R1275Q Neoepitopes Using ProTECT

**Figure 1:**
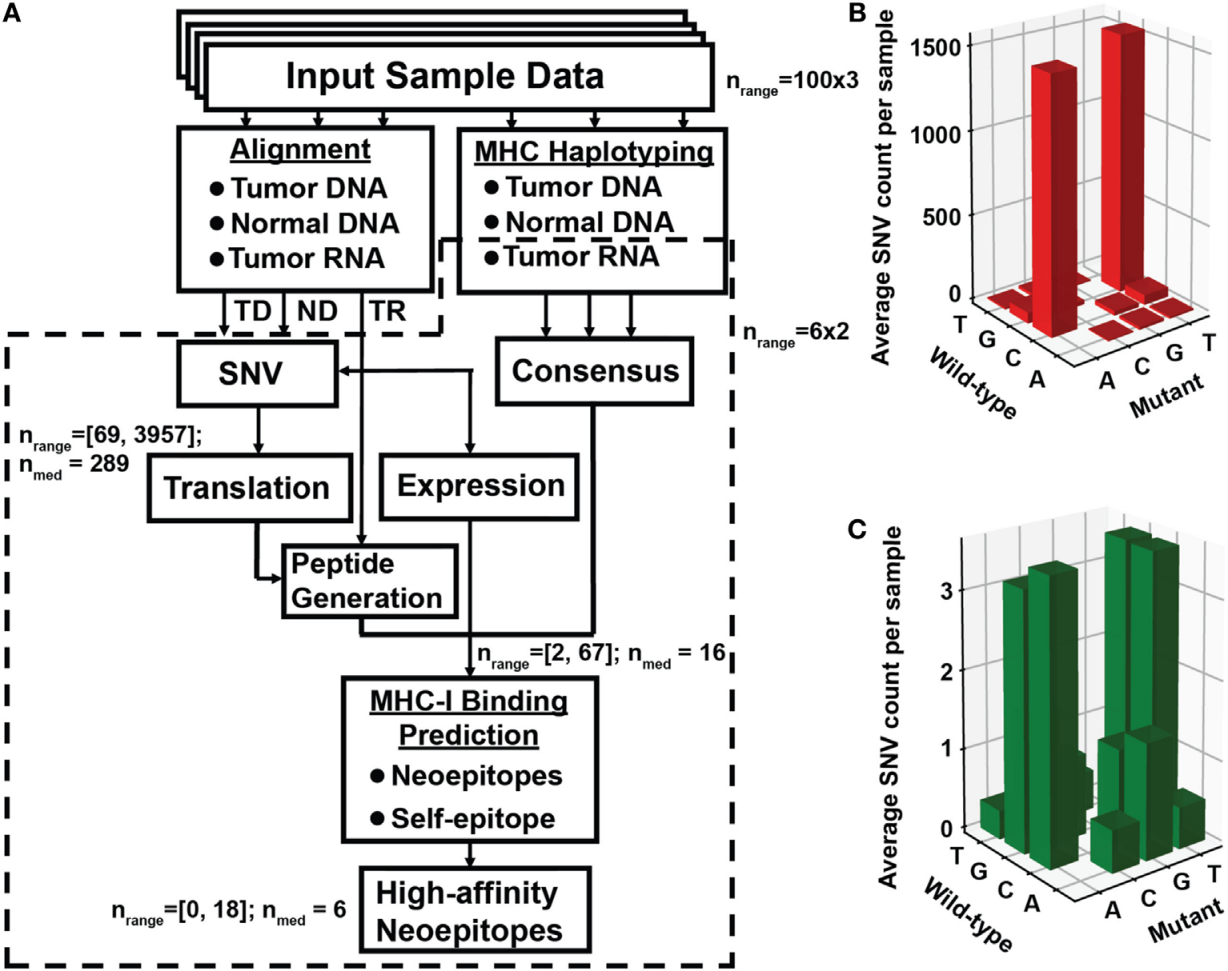
Identification of neoantigen targets using the ProTECT pipeline. **(A)** Flowchart indicating each step of the ProTECT pipeline. Input FASTQs trios per sample ultimately give rise to MHC haplotyping and provide a list of candidate neoepitopes for each sample. Abbreviations: TD and ND, tumor and normal DNA, respectively; TR, tumor RNA. Predicted tumor–normal single-nucleotide variants (SNVs) are filtered during peptide generation, and again at neoepitope prediction. *n*_range_: the range of SNV calls that make it past a certain step; *n*_med_: median number of calls. The primary:relapse pairs were run through a smaller modified version of the pipeline that started directly from mutations curated from Eleveld et al. (16). Panels **(B,C)** show the TARGET neuroblastoma cohort OxoG mutation level. Before filtering for OxoG artifacts, we see a predominance of C>A/G>T mutants **(B)**, whereas after filtering we see a marked reduction in the total number of mutations and a more balanced nucleotide substitution rate **(C)**.

A reduced version of our software, ProTECT (Figure 1), was initially run on a batch of six primary:relapsed NBL sample pairs from the TARGET cohort. We find at least one neoepitope-generating mutation persisting in the relapsed tumor for five of six patients (Table S1 and Supplementary Data S1 in Supplementary Material). Among these are two well-known hotspot mutations, *NRAS* Q61K and *ALK* R1275Q (Table S1 in Supplementary Material). We predicted two HLA-B*15:01-restricted decamer (MAQDIYRASY and AQDIYRASYY) and one nonamer (AQDIYRASY) neoepitopes arising from *ALK* R1275Q in sample TARGET-30-PARHAM. The predicted binding affinities are better than 0.55, 0.85, and 2.1%, respectively, relative to all peptides in a background training set (the top 5% ranked peptides are considered true binders by our method). While the peptide beginning at M1273 is predicted to be the top binder, the two epitopes beginning at A1274 are more promising from an immunological perspective since they are predicted to be significantly better binders to HLA-B*15:01 than their parental self-antigens ARDIYRASYY (10.75 percentile score) and ARDIYRASY (35 percentile score).

Using the full version of the ProTECT software (manuscript in preparation), we expanded our study to 100 primary NBL samples in the TARGET cohort to collect more complete statistics on *ALK* R1275Q-derived neoepitopes, and to identify other recurrent neoepitopes in NBL (Table S1 and Supplementary Data S2 and S3 in Supplementary Material). We identify four additional samples harboring *ALK* R1275Q (TARGET-30-PANWRR, -PANXJL, -PAPTFZ, and -PAPTLV). None of these samples express HLA-B*15:01, but sample PAPTFZ displays a close relative, HLA-B*15:03 that is predicted to bind ARDIYRASYY and ARDIYRASY with scores of 2.2 and 4.7%, respectively. Two samples (PANXJL and PAPTLV) express the high-frequency HLA-A*02:01 (20% in Caucasian populations), where an *ALK* R1275Q nonamer (GMAQDIYRA) is predicted to bind HLA-A*02:01 with a 1.4% score.

All but six of the 100 samples harbor one or more non-synonymous neoepitope with low percentile scores for at least one expressed HLA allele. Among these we identify other recurrent mutations, including the *ALK* mutation F1174L/I/C, present in 3/2/1 samples, respectively, and a *ZNF717* mutation (Q716), present in three samples (Table S1 in Supplementary Material). One sample in the cohort expresses *NRAS* Q61K, an activating mutation commonly found in melanoma, thyroid, and colorectal cancers. Finally, the *NRAS*-derived neoepitope ILDTAGKEEY arising from a single mutation (Q61K) is predicted to bind the common HLA-A*01:01 allele with a statistically significant score of 0.35%. Notably, the same HLA-A*01:01/ILDTAGKEEY interaction identified by our method has been previously shown to elicit a specific T cell response using a melanoma cell line (21).

### *ALK* Tumor Neoantigens Form p/MHC Complexes with Distinct Stabilities *In Vitro*

**Figure 2:**
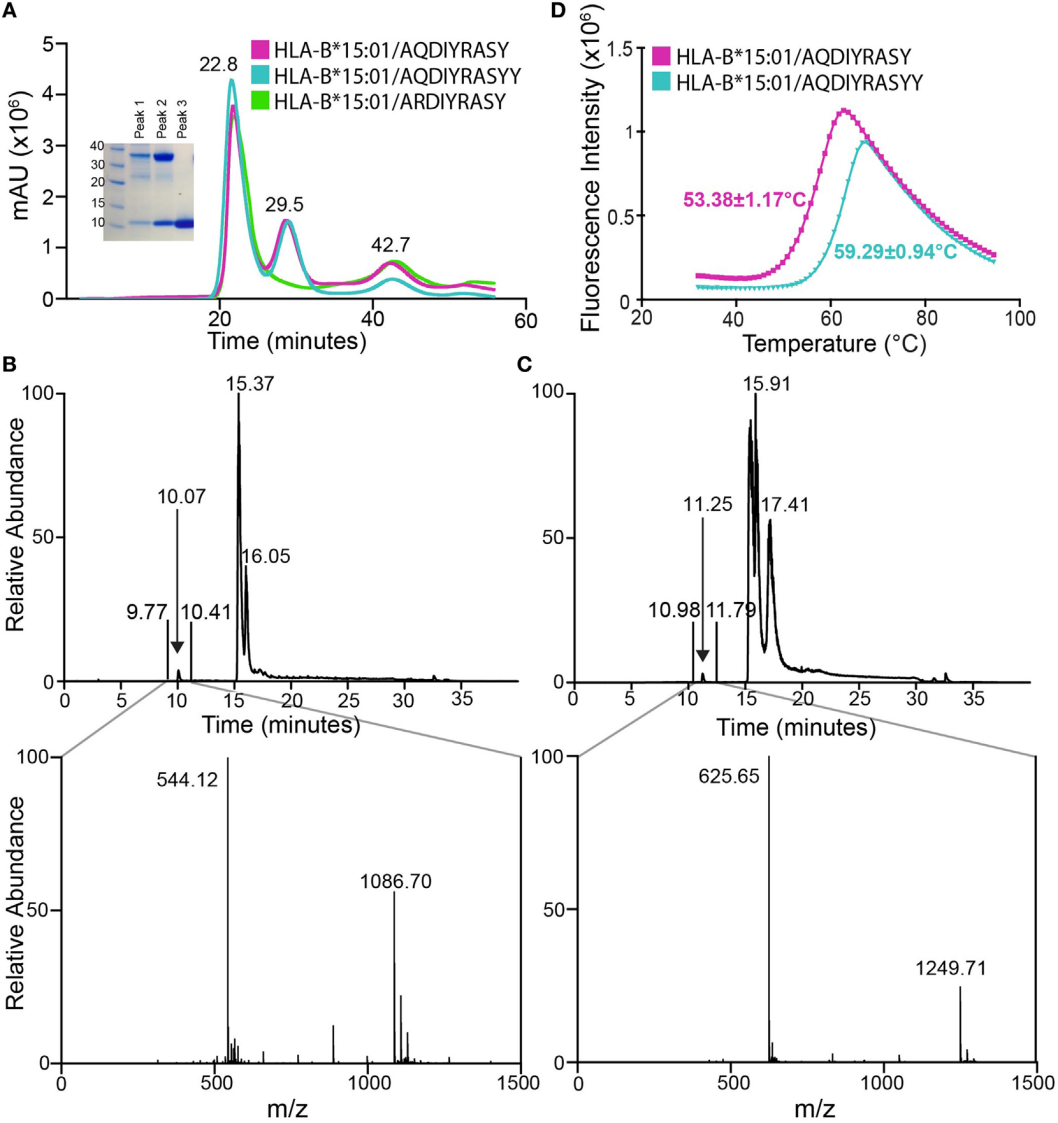
Association of anaplastic lymphoma kinase neoepitopes with recombinant HLA-B*15:01 *in vitro*. **(A)** Size exclusion chromatography (SEC) traces of MHC samples refolded with nonamer (magenta), decamer (teal), or self (green) peptides. Purification was performed on a HiLoad 16/600 Superdex 75 pg column at a flow rate of 2 mL/min. Eluted fractions were probed using SDS-PAGE analysis followed by Coomassie staining (left) and show expected molecular weights for HLA-B*15:01 (32.4 kDa) and β_2_m (11.8 kDa). Further analysis reveals SEC peak identities as protein aggregate (22.8 min), p/MHC complex (29.5 min), and free β_2_m (42.7 min). Attempts at refolding HLA-B*15:01 with the self-peptide did not produce a p/MHC complex (green curve, lack of 29.5 min peak). LC–MS analysis of purified nonamer **(B)** and decamer **(C)** HLA-B*15:01 complex samples. The top panel shows the chromatogram trace of each complex, while the bottom panel is the average relative abundance for the time interval between 9 and 11 min, showing the presence of either the nonamer (observed mass 1,086.70 Da; expected mass 1,086.17 Da) or the decamer peptide (observed mass 1,249.71 Da; expected mass 1,249.35 Da) captured in the MHC peptide-binding groove. **(D)** Differential scanning fluorimetry shows that the decamer-bound MHC complex (teal) has an increased thermal stability of 59.3°C relative to the 53.4°C *T*_m_ observed for the nonamer-bound MHC complex (magenta).

The results obtained from ProTECT analysis provide a range of therapeutically relevant neoantigen/HLA interactions to validate and characterize using biophysical and structural methods. Given the extensive genetic evidence supporting a role for *ALK* mutations in NBL tumors (15, 17), we chose to pursue further the interaction between *ALK* R1275Q and HLA-B*15:01. We prepared recombinant HLA-B*15:01 bound to the two *ALK*-derived neoantigens, a nonamer (AQDIYRASY) and a decamer (AQDIYRASYY). As a control, we attempted to prepare HLA-B*15:01 with the self-antigen (ARDIYRASY), which is predicted to have a >10-fold reduced binding affinity for the HLA. Peptide/MHC samples were refolded from purified *Escherichia coli* inclusion bodies in the presence of 10-fold molar excess peptide using standard methods and purified by size exclusion chromatography (SEC) (22). SEC traces of the nonamer and decamer samples show three distinct peaks corresponding to protein aggregate (22.8 min), p/MHC complex (29.5 min), and free β_2_m (42.7 min) (Figure 2A). Notably, the sample refolded using the self-antigen peptide shows only two peaks in the chromatogram, none of which contain non-aggregated p/MHC molecules (Figure 2A, green trace), further suggesting that the affinity of the self-antigen is insufficient to promote the formation of a stable complex with the HLA.

To confirm the presence of the neoepitopes in the two MHC samples, we performed liquid chromatography–mass spectroscopy (LC–MS). LC–MS reveals a high relative abundance of the correct peptide in each sample, with observed masses of 1,086.70 and 1,249.71 Da, which agree well with the expected masses of the nonamer and decamer, respectively (Figures 2B,C). Thus, we confirm binding of the two tumor neoepitopes to recombinant HLA-B*15:01 prepared through *in vitro* refolding. To further characterize the resulting p/MHC molecules, we used a differential scanning fluorimetry (DSF) assay, which can accurately assess kinetic stability. According to this technique, properly folded class I p/MHC complexes show melting temperatures (*T*_m_) from 37 to 63°C, which correlate with predicted IC_50_ values in the micromolar to nanomolar range (23). Here, both neoantigen p/MHC samples show a clear unfolding transition with a highly reproducible *T*_m_ of 53.4°C for the nonamer and 59.3°C for the decamer complex (Figure 2D), suggesting that the decamer forms a higher affinity complex with HLA-B*15:01. Such a difference in thermal stabilities of the p/MHC complexes together with previous observations that peptide length influences its conformation within a fixed-length groove is consistent with a hypothesis that the two peptides are displayed *via* distinct binding modes, as previously reported for nonamer and decamer peptides sampling unique conformations within an MHC groove (24).

### Structural Plasticity within the MHC Peptide-Binding Groove Enables Distinct Neoantigen Conformations

**Figure 3:**
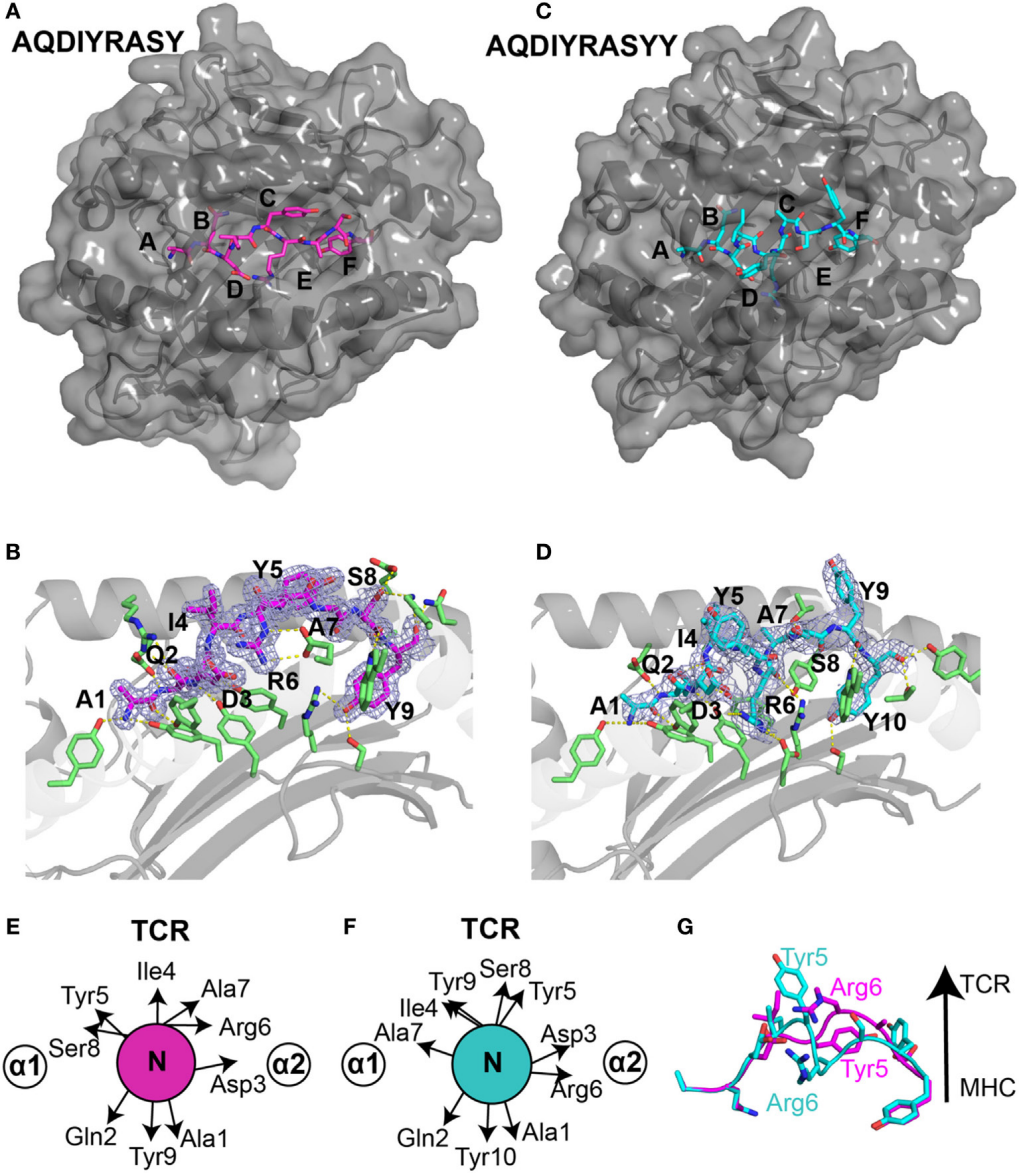
Structural differences in *ALK* neoepitope displayed by HLA-B*15:01. X-ray structures of the **(A)** nonamer peptide (PDB ID 5TXS), shown as magenta sticks and **(C)** decamer peptide (PDB ID 5VZ5), shown as cyan sticks embedded into the groove of HLA-B*15:01 molecule. The canonical peptide-binding pockets in HLA groove are indicated with letters. **(B)** Nonamer peptide (magenta sticks) and **(D)** decamer peptide (cyan sticks) with 2*F*_o_ − *F*_c_ electron density maps contoured at 1.2 σ within the groove of HLA-B*15:01. Yellow dashes represent polar contacts between the peptide and selected MHC residues (green sticks). Side-chain orientation of the **(E)** nonamer peptide and **(F)** decamer peptide as viewed from the top axis of the peptide highlighting the placement of different residues. **(G)** Structural superposition heavy backbone atoms of the bound nonamer (magenta sticks) and decamer (cyan sticks) neoepitopes (all-atom RMSD of 4.0 Å) reveal distinct T cell receptor (TCR)-interacting residues between the two neoantigens.

To elucidate the structural basis underlying the distinct stabilities observed for the two *ALK* neoepitopes and to further characterize peptide features displayed to TCRs we solved the X-ray structures of the nonamer (HLA-B*15:01/β_2_m/AQDIYRASY) (PDB ID 5TXS) and the decamer complex (HLA-B*15:01/β_2_m/AQDIYRASYY) (PDB ID 5VZ5). The nonamer complex crystallized in the P2_1_2_1_2_1_ space group at a resolution of 1.7 Å, while the decamer complex crystallized in the P6_1_22 space group at a resolution of 2.6 Å (Table S2 in Supplementary Material). The nonamer peptide adopts a canonical extended conformation promoted by the N-terminal (Ala1, Gln2) and C-terminal (Tyr9) anchors, which are deeply embedded within A/B, and F-pockets of the HLA groove (Figures 3A,B), respectively. This anchoring results in a “curved” conformation, where the backbone of residues from Asp3 to Ser8 is pushed toward the upper part of the groove while the remaining residues are maintained within the C, D, and E pockets (Figure 3B). A survey of previously deposited HLA-B*15:01-restricted antigens in the PDB (LEKARGSTY derived from Epstein–Barr virus, PDB ID 1XR8; ILGPPGSVY derived from human ubiquitin-conjugating enzyme-E2, PDB ID 1XR9; VQQESSFVM derived from SARS coronavirus, PDB ID 3C9N) reveals other nonamer epitopes consistently in extended conformations (25, 26), in agreement with the conformation of the *ALK* nonamer neoepitope in our X-ray structure (Figures S4A–D in Supplementary Material). Furthermore, the overall architecture of the B*15:01-binding groove is similar between the different structures with heavy atom backbone RMSDs of less than 1 Å (Figure S4E in Supplementary Material). Comparison between the peptide amino acid sequences reveals excellent agreement with the established HLA-B*15:01-binding motifs, where LMQ/AEISTV and FY/LM are preferred/tolerated in anchor positions 2 and 9, respectively (Figures S4F,G in Supplementary Material). Thus, the X-ray structure of our *ALK*-derived nonamer neoepitope is consistent with established structural features in the PDB, suggesting a trend where the peptide backbone conformation is defined by its length and anchor motifs.

Generally, peptides of length greater than nine amino acids either bulge further out of the binding groove or form a “zig-zag” conformation (27). However, in our decamer complex structure (Figures 3C,D), the peptide adopts a short 3_10_ helical backbone conformation from Ile4 to Ala7, as confirmed by an inspection of φ/ψ backbone dihedral angles (Figure S1A in Supplementary Material). Notably, while the N-terminal anchor residues are identical in the nonamer and decamer peptide, Tyr10 of the decamer replaces Tyr9 of the nonamer as the C-terminal anchor residue in a similar conformation (Figures 3B,D). The accommodation of a longer peptide sequence within the fixed-size MHC groove is thus achieved through the formation of a more compact 3_10_ helix for the decamer, relative to the extended nonamer backbone. In addition, the 3_10_ helix buries Arg6 further into the MHC groove and creates an amphipathic structure where Ile4, Tyr5, Ser8, and Tyr9 are oriented toward the solvent (Figure 3D). A structural superposition of the nonamer and decamer peptides (2.7 Å backbone heavy atom RMSD) highlights the changes in residues that are oriented toward the solvent, suggesting that the two epitopes display very different surface features for interactions with TCRs (Figures 3E–G).

The compaction of the peptide backbone in the decamer structure is accompanied by structural adaptations of MHC residues in the peptide-binding groove. In particular, in the decamer complex the HLA α_2_ helix undergoes a significant widening involving a 5.1 Å displacement of the Cα atom of Arg151. This movement is driven by a change in orientation of Arg151, which points toward the solvent in the nonamer versus toward the groove in the decamer complex (Figures S2A,B in Supplementary Material), and the burying of Arg6 further toward the floor of the groove. Thus, the addition of a C-terminal Tyr in the peptide sequence drastically alters the tertiary structure of the HLA complex, driven by a widely different peptide conformation that can be accommodated through conformational plasticity within a malleable MHC groove.

Key structural parameters extracted from our crystallographic analysis provide insights into the increased stability of the decamer/HLA complex. Notably, the buried surface area (BSA) between HLA-B*15:01 and the decamer peptide is 1,986 Å^2^, relative to 800 Å^2^ in the nonamer structure. To further dissect different structural features for their contributions to p/MHC stability, we analyzed all polar (hydrogen bonds, salt bridges, and electrostatic interactions) and hydrophobic interactions involving HLA residues (Figure S5 and Table S3 in Supplementary Material). Specifically, the decamer peptide forms additional intra-peptide hydrogen bonds as a result of the more compacted 3_10_ helix conformation. In addition, the decamer participates in 25 polar and 21 hydrophobic interactions with the MHC residues, while the nonamer forms 26 polar but only 11 hydrophobic interactions with the groove (Figure S5A in Supplementary Material). Specifically, Asp3 and Arg6 of the decamer peptide extend further into the groove, forming additional contacts with HLA side chains (Figures S5B,C in Supplementary Material). Our structural analysis suggests that an increase in the total number of intra-peptide hydrogen bonds and hydrophobic packing interactions, consistently with an increase in BSA brought on by the more compact 3_10_ helical conformation, leads to an improved stability of the decamer complex, as confirmed independently by our DSF experiments (Figure 2B).

### Structural Exclusion of the Self-antigen from the HLA-B*15:01 Groove

To further evaluate the potential immunogenicity of the *ALK* R1275Q neoepitopes, we compared their affinity for HLA-B*15:01 relative to the self-peptide (ARDIYRASY). Formation of a stable HLA complex displaying the self-peptide would compromise the therapeutic relevance of any related neoantigen, due to immune tolerance mechanisms that limit the repertoire of responsive T cells. Preliminary attempts to refold HLA-B*15:01 using a synthetic nonamer peptide with the parental *ALK* sequence did not result in efficient p/MHC formation, suggesting low binding affinity, likely in the micromolar range (Figure 2A, green trace). To further explore the basis of this exclusion we performed structural modeling of the self-peptide/HLA-B*15:01 complex, using our solved X-ray structure of the nonamer complex as a template. We find that performing the reverse Gln to Arg substitution leads to steric hindrance between the longer Arg2 side chain and residues of the MHC-binding groove (Figure S6A in Supplementary Material). Despite a careful consideration of all possible Arg side-chain rotamers, significant clashes remain with Ser67 on the α_1_ helix, as well as with Ala24, Met45 on the floor of the MHC groove (Figure S6A in Supplementary Material). As expected from the conservation of peptide residue anchors in the A- and B-pockets, we observe similar clashes when the self-decamer is modeled with HLA-B*15:01. By contrast, the neoepitope Gln2 side chain fits well into the B-pocket, forming an additional hydrogen bond Tyr9 from the HLA heavy chain (Figure S6B in Supplementary Material, cyan dotted line). Finally, we performed detailed structure modeling calculations using simultaneous optimization of the peptide backbone in addition to the side-chain degrees of freedom and ranked the calculated affinities of the three peptides for HLA-B*15:01 according to a physically realistic energy function (28). The self-antigen complexes yield the least favorable binding energies, followed by the nonamer, and finally the decamer complex (Figure S3 in Supplementary Material). Thus, our structural analysis is highly consistent with our *in vitro* results, i.e., that the self-peptide is excluded from binding, in sharp contrast with the nonamer and decamer neoepitopes which form tight complexes with the HLA.

### Evaluating the HLA-Binding Repertoire Using Comparative Modeling Calculations

**Figure 4:**
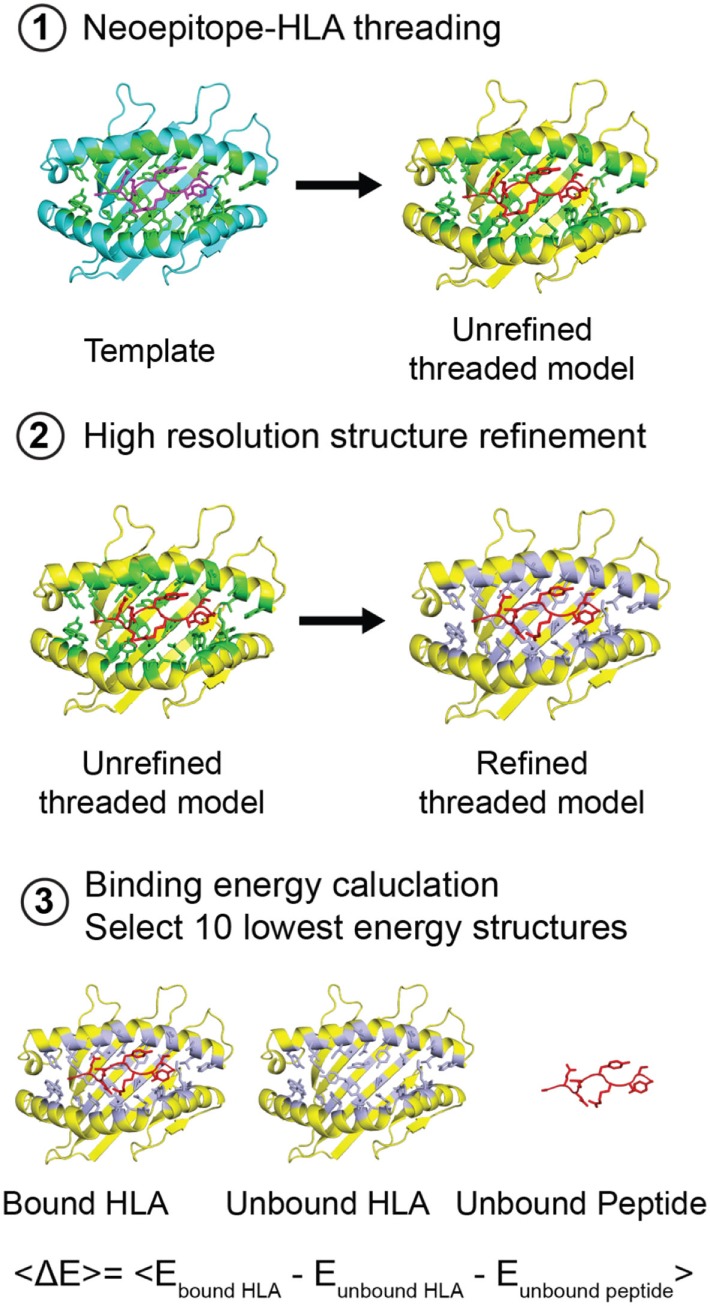
Structure-based modeling of neoepitope/human leukocyte antigen (HLA) interactions. Step 1: A template (blue) peptide/HLA complex (X-ray structure) is provided to generate a threaded model with the same peptide and different HLA alleles (yellow). HLA residues in the groove within 3.5 Å of the peptide are colored green. Step 2: Models are refined by energy minimization and side-chain repacking of groove and peptide residues (gray). Step 3: The average peptide-binding energy is determined by subtracting the energy of the unbound HLA and unbound peptide from the energy of the peptide bound HLA. <E> represents the average binding energy. The top 10 lowest energy structures are compared with determine a consensus model.

**Figure 5:**
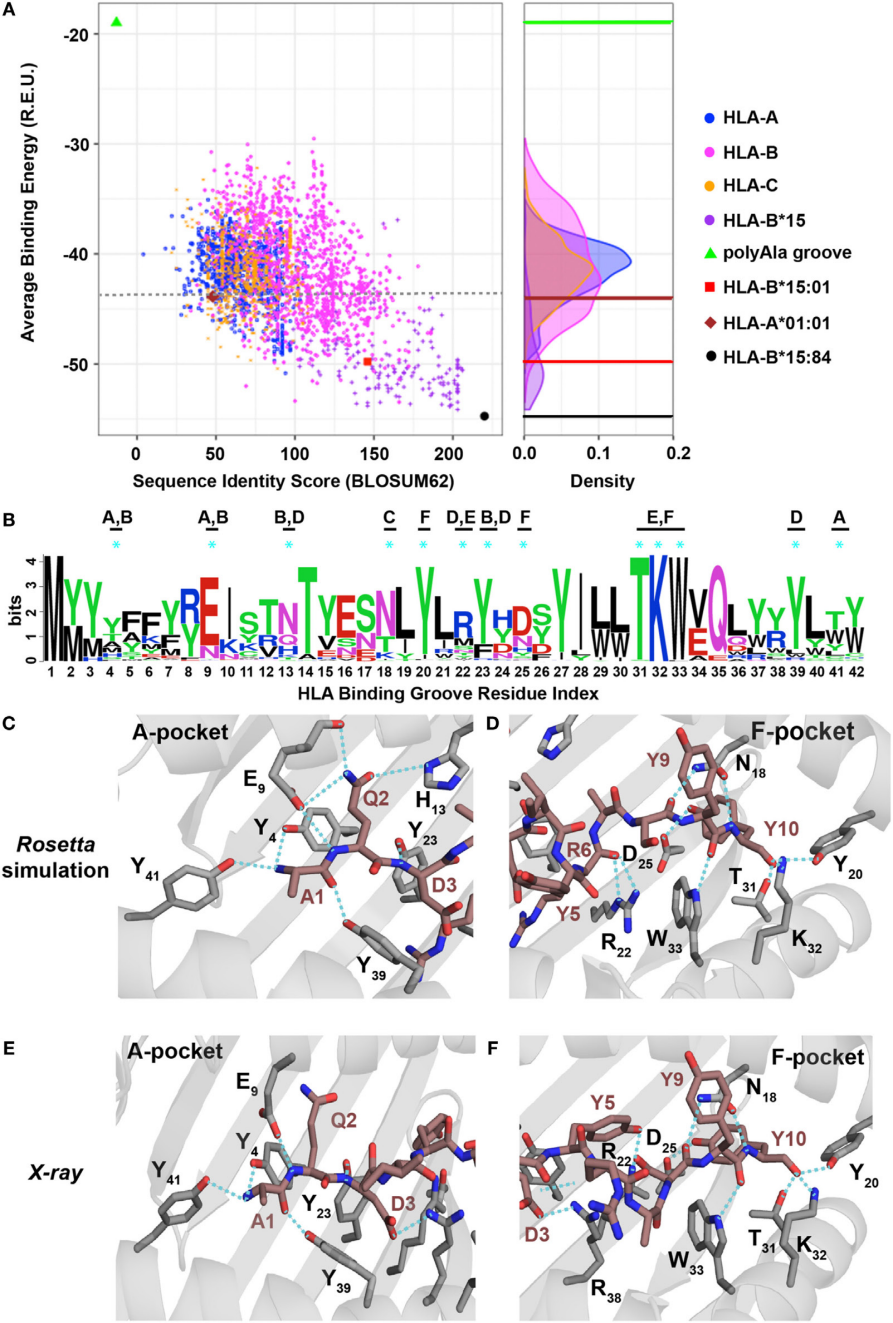
Evaluating the human leukocyte antigen (HLA)-binding repertoire of *ALK* decamer AQDIYRASYY using *Rosetta* structure-based modeling. **(A)** *Rosetta*-binding energies calculated from structure modeling of 2,904 unique HLA alleles from the IPD-IMGT/HLA Database (29), for the *ALK* neoepitope decamer (AQDIYRASYY) plotted as a function of sequence similarity to the top binding allele, HLA-B*15:84 (black circle). The binding energy of decamer in our HLA-B*15:01 X-ray structure is shown as a reference (red square). A negative control was performed with a mock HLA allele where all residues in the binding groove were replaced with Ala (polyAla groove, green triangle), which shows high-binding energy. The corresponding distribution of the HLA alleles on the binding energy landscape is captured in the density plot shown on the right. Sequence identity scores were calculated using the BLOSUM62 (33) matrix. Abbreviation: R.E.U., *Rosetta* energy units. **(B)** Kullback–Leibler sequence logo derived from multiple sequence alignment using ClustalOmega of peptide-binding groove residues from all the HLA alleles that exhibit better binding energies than HLA-A*01:01 (brown diamond), indicated with a gray dotted line in panel **(A)**. MHC residues with polar contacts to the peptide are denoted with a cyan asterisk with corresponding MHC pocket noted. **(C,D)** Threaded structural model of HLA-A*01:01 displaying decamer peptide. Polar contacts between the MHC groove (gray sticks) and peptide (brown sticks) are shown with cyan dotted lines in the A-, B-, and D-pockets **(C)** or C-, E-, and F-pockets **(D)**. The residue index for each interacting MHC residue is denoted with the corresponding number from panel **(B)** using subscripts. Peptide residues (non-indexed) are labeled without subscripts. Panels **(E,F)** show polar contacts observed in the A-pocket **(E)** and F-pocket in the X-ray structure of HLA-A*01:01/AQDIYRASYY (PDB ID 6AT9) between the peptide (brown sticks) and residues in the MHC groove (gray sticks). The residue index for each interacting MHC residue is denoted with the corresponding number from panel **(B)** using subscripts. Peptide residues (non-indexed) are labeled without subscripts.

A patient’s HLA haplotype plays a major role in determining the outcome of targeted cancer immunotherapies. Therefore, toward expanding the range of individuals that could mount a T cell response to *ALK* R1275Q neoepitopes, we evaluated the potential of other HLAs to display the two peptides *in silico*. Here, we developed and applied a high-throughput approach which exploits the availability of our high-resolution X-ray structures for the two neoepitopes to simultaneously predict peptide/HLA interactions and surface features of peptide residues poised for interactions with TCRs. First, we selected a non-redundant set of 2,904 HLA alleles (885 HLA-A, 1,405 HLA-B, and 614 HLA-C unique sequences) from the EMBL-EBI database (29). We then carried out detailed *Rosetta* comparative modeling calculations for each allele, using our experimentally determined HLA-B*15:01 structures for the nonamer and decamer ALK peptides as templates (Figure 4). In contrast to previous structure-based peptide/HLA modeling methods which use a flexible peptide docking approach (30–32), we used a fixed-peptide backbone threading approach followed by energy minimization of the interacting peptide and HLA residues to drastically confine the docking degrees of freedom. Our approach was motivated the observation that the peptide backbone conformation shows minimal variance (less than 1.5 Å RMSD) in all nonamer/HLA-B*15:01 structures reported in the PDB (Figure S4E in Supplementary Material). Using this strategy, we extracted highly reproducible binding energies for both the nonamer and decamer peptides, which are maintained in extended and 3_10_ helical conformations, respectively, in the resulting models (Figure S7 in Supplementary Material). As expected, the HLA-B*15 alleles rank systematically among the top binders, indicating a high degree of groove complementarity to both peptides (Figure 5; Figure S8A in Supplementary Material, purple). Among those, the HLA-B*15:84 allele shows the lowest binding energy for the decamer (Figure 5A, black circle), whereas the HLA-B*15:107 allele shows the lowest binding energy for the nonamer (Figure S8A in Supplementary Material, black circle). A total of 116 HLA alleles from all A, B, and C types exhibit lower binding energies for both the nonamer and decamer peptides than our initial HLA-B*15:01 structural templates (Figure 5A; Figure S8A in Supplementary Material, red square), suggesting the potential for a broader HLA display repertoire.

To elucidate a sequence bias for specific residues in the HLA-binding groove that consistently yield more favorable interactions with the two peptides, we analyzed the average binding energy as a function of sequence identity score (33), calculated relative to the best binding allele for each peptide (Figure 4). As a negative control, we computed the binding energy for a mock HLA allele in which all residues in the MHC-binding groove are mutated to Ala. As expected, the mock polyAla HLA exhibits a low binding affinity (i.e., high-binding energy) to the peptide and is distant from the best binding allele (Figure 5; Figure S8A in Supplementary Material, green triangle, top left). We observe an evident correlation between the computed binding energies and sequence similarity to the top binder. Our approach additionally allows us to decompose residue specific contributions to overall binding energy for each peptide–HLA combination. We find a clear trend for both the nonamer and decamer peptides with a set of HLA alleles where a bulk of the binding energy is provided by the “anchor” positions (Figures S11A,B in Supplementary Material). By contrast, the mock polyAla HLA exhibits considerably higher binding energy across the entire peptide length (Figures S11A,B in Supplementary Material). To elucidate key sequence features that allow the peptides to be accommodated in the MHC groove, we derived a sequence profile among good binders for the two neoepitopes. Such features are highlighted in the Kullback–Leibler sequence logo, which reveals preferred residues in the HLA peptide-binding groove (Figure 5B; Figure S8B in Supplementary Material). According to this metric, highly invariant residues in the MHC-binding groove should play an essential role in mediating peptide/MHC interactions, as they are consistently observed in HLA alleles that exhibit high affinity binding. A close inspection of our structural models for the nonamer and decamer bound to a common allele in our data set, HLA-A*01:01, reveals similar polar contacts, primarily in the A-, B-, and F-pockets, that correlate well with the positions of invariant MHC residues (Figures 5C,D; Figures S8C,D in Supplementary Material). Specifically, both the nonamer and decamer C-terminal anchors employ a similar interaction pattern in the F-pocket with conserved Thr, Lys, Trp, and Tyr residues of the MHC (Figures 5B,D; Figure S8B,D in Supplementary Material).

To test the validity of our structure-based simulations, we performed *in vitro* refolding of the *ALK*-derived nonamer and decamer peptides with HLA-A*01:01. This allele was chosen because it is a high-frequency allele in multiple populations worldwide and has been previously shown to form stable recombinant p/MHC complexes for structural characterization (34). As observed in our previous experiments with HLA-B*15:01 (Figure 2A), refolding of HLA-A*01:01 with decamer or nonamer peptide results in a stable p/MHC complex (Figures S8E and S9A in Supplementary Material). Further characterization of the purified complex reveals a thermal stability of 47.9°C for the decamer (Figure S9B in Supplementary Material) and 46.7°C for the nonamer (Figure S8F in Supplementary Material), suggesting that both *ALK* neoepitopes have a lower affinity for HLA-A*01:01 compared with HLA-B*15:01 (Figure 2D, 59.3°C), consistently with our binding energy calculations (Figure 5A; Figure S8A in Supplementary Material). Although certain HLA and H2 MHC alleles have been previously reported to yield partially folded, peptide-free molecules with measurable thermal stabilities (35), control refolding experiments performed without peptide for each of our HLA alleles failed to yield a stable complex. Finally, to conclusively test the atomic features predicted by our simulations, we determined the X-ray structure of decamer complex HLA-A*01:01/β2m/AQDIYRASYY (PDB ID 6AT9). The decamer complex crystallized in the P32_2_21space group at a resolution of 2.9 Å (Table S2 in Supplementary Material). Inspection of crystallographic φ/ψ dihedral angles reveals that the peptide backbone also adopts a short 3_10_ helix conformation when bound to HLA-A*01:01, suggesting that the peptide length is the main determinant of its conformation in the groove, and further justifying our fixed-backbone modeling approach (Figure S9F in Supplementary Material). The peptide conformation in the X-ray structure shows excellent agreement with our *Rosetta* model (1.1 backbone heavy atom RMSD), with several high-resolution features predicted by the model are confirmed by the X-ray, including polar contacts within both the A- and F-pockets of the MHC groove (Figures 5C–F; Figure S12 in Supplementary Material). Specifically, the side-chain hydroxyl group of the peptide Tyr10 is in contact with the same Tyr, Lys, and Trp side-chain atoms from the F-pocket (Figures 5D,F). Finally, in comparison with the X-ray structure of the same peptide bound to HLA-B*15:01, the side chain of Arg6 is flipped outwards from the groove when bound to HLA-A*01:01 altering the peptide surface displayed to TCRs (Figures S9D,E in Supplementary Material). Thus, our independent X-ray structure corroborates the trend observed in our structure-based binding energy simulations and further supports the potential for other HLA molecules to display the recurrent *ALK* neoepitopes with unique TCR interaction properties.

### The Two *ALK*-Derived Neoepitopes Are Recognized by CD8+ T Cells

Given the unique conformations and surface features observed for the nonamer and decamer peptides, we sought to determine whether the two altered-self (i.e., mutated) neoantigens could be recognized by CD8+ T cells using a MHC tetramer staining assay followed by multichannel flow cytometry analysis. We hypothesized that an HLA-matched donor would be able to recognize altered-self neoepitopes in the periphery, as long as the peptide adopts a conformation that can potentiate interactions with TCRs. To test this, we acquired PBMCs from two HLA-B*15:01-matched healthy donors. For each peptide, we performed a double staining experiment using HLA-B*15:01 tetramers conjugated with allophycocyanin (APC) or phycoerythrin (PE), toward identification of T cells that recognize each neoepitope. Final cell sorting using fluorescence-based detection results in identification of double positive populations with a total of 0.012% CD8+ T cells reactive to the nonamer (Figure S13A in Supplementary Material), and 0.024% reactive to the decamer epitope (Figure S13B in Supplementary Material). Notably, these findings were very similar between two independent staining experiments using PBMCs from individual donors (Figure S13C in Supplementary Material). As a control, we additionally performed staining experiments using tetramers made for HLA-B*15:01 complexed with an immunodominant SARS coronavirus-derived epitope (VQQESSFVM) (26). For double staining experiments with HLA-B*15:01/SARS tetramers, we observe double positive populations corresponding to 0.007 and 0.014% reactive CD8+ T cells (Figure S13C in Supplementary Material). Finally, simultaneous staining experiments using nonamer/HLA-B*15:01-PE and decamer/HLA-B*15:01-PE tetramers did not uncover populations of CD8+ T cells that recognized both epitopes (Figure S13C in Supplementary Material). Thus, CD8+ T cells are able to recognize both neoepitopes with nominal frequencies that are comparable to that of a known immunodominant epitope.

## Discussion

Immunotherapies that stimulate the immune system to attack tumors, including immune checkpoint blockade and adoptive T cell therapies, have achieved spectacular results in tumor types with high mutational burden, such as melanoma (36). However, their utility in tumors with lower mutational burden, such as those that occur in pediatric cancers, is less clear (37). The development of more targeted T cell based immunotherapies to treat cancer relies on understanding the molecular basis of neoepitope display on tumor cells, in addition to the initiation and regulation of cytotoxic CD8+ T cell responses (38). A current roadblock in the development of robust approaches across patients is that the HLA locus is extremely polymorphic, and an individual’s exact HLA haplotype sculpts the repertoire of epitopes displayed to the immune system (5). Moreover, the identification of therapeutically relevant antigens in tumors remains extremely challenging and is further complicated by the fact that a single HLA allele can potentially bind 10^3^–10^6^ distinct peptide epitopes (39). Traditionally, *in vitro* measurements of affinities between an MHC and a potential antigen were achieved by equilibrium dialysis (35) and fluorescence polarization experiments (40). More recent approaches allow for a global evaluation of the entire peptide repertoire, using mass spectroscopy of MHC complexes extracted from cell lines expressing a single HLA allele followed by bioinformatics analysis (41). Robust alternative strategies to identify and characterize neoepitope/HLA complexes with desired T cell recognition features would significantly bolster the progress of targeted T cell therapies against cancer.

We have recently developed ProTECT, a fully automated and freely available tool for predicting expressed neoepitopes based on the somatic mutations present in tumor samples. In NBL, a common pediatric cancer, ProTECT analysis identifies a range of intriguing predicted high affinity neoepitope–HLA targets that should be examined in future studies, such as *NRAS*:Q61K—HLA-A*01:01 (Table S1 in Supplementary Material), including the *ALK* neoepitopes examined in detail here. Typically, Immune Epitope Database (IEDB)-based binding prediction methods are biased towards nonamer peptides due to limited number of datasets for peptide/MHC-binding affinity measurements of shorter or longer peptide lengths (19, 20). Moreover, affinity thresholds for binding based on IC_50_ values are HLA allele specific and range from 60 to 950 nM (42), which could result in false negative predictions where weak binding epitopes that may be immunogenic are not considered. We attempt to normalize for these limitations in ProTECT by using a suite of predictors trained on combined and/or allele-specific datasets that consider a range of epitope-binding affinities. In our analysis, we find that 90% of the NBL samples have one or more predicted high affinity neoepitope–HLA targets. We sought to characterize the nature of the p/MHC interactions resulting from the relatively common *ALK* R1275Q mutation, to lay the groundwork for developing a targeted immunotherapy for it, and to develop a pipeline for evaluating other promising tumor neoepitopes. Toward these goals, we have elucidated the structural characteristics underlying the *in vitro* stability and presentation of two *ALK* R1275Q-derived nonamer and decamer epitopes where the corresponding self-peptide does not bind to the same HLA groove. We additionally developed and applied a high-throughput comparative modeling approach to identify additional HLA alleles that could display the two neoepitopes and predict their structures with high accuracy, toward understanding the link between peptide surface features and interactions with TCRs. Finally, we examined the potential for the ALK-derived neoepitope/HLA complexes to activate an immune response by analyzing CD8+ T cell recognition from HLA-B*15:01-matched donors.

The exact conformation and dynamic features of the peptide within the MHC-I-binding groove are known to play pivotal roles in recognition by CD8+ T cells, by dictating MHC/peptide-binding affinity, stability on the cell surface and cross-reactivity of interactions with specific TCR molecules (43, 44). Our X-ray structures reveal an extreme case of such conformational plasticity, in which the addition of a single C-terminal Tyr in the neoantigen sequence (AQDIYRASY to AQDIYRASYY) significantly alters the peptide conformation (Figures 3E–G). This dramatic change relative to the canonical extended structure is highlighted by the formation of a 3_10_ helix spanning residues Ile4 to Ala7 (Figure S1 in Supplementary Material) and provides a link between peptide conformation and HLA complex stability. Specifically, the 3_10_ helix leads to an increase in BSA and number of molecular interactions between the peptide and HLA side chains (Figure S5 in Supplementary Material), in agreement with its increased thermal stability (Figure 2D). We additionally observed changes in the HLA groove, including a displacement of the α2 helix that undergoes a significant widening involving a 5.1 Å movement of the Cα atom of Arg151 (Figure S2A,B in Supplementary Material). Our results further support the importance of the α2 helix, which participates in a myriad of immune processes, such as chaperone-mediated peptide loading/editing (45), allele-specific antigen presentation (46), and TCR recognition (47), in the context of conformational plasticity of the MHC groove to accommodate epitopes of varying length. Finally, structure modeling of the self-antigen sequence, in agreement with *in vitro* refolding experiments, shows a sharp contrast in stability relative to the neoantigens due to an Arg anchor that cannot be accommodated on either an extended or helical backbone conformation. Our results provide a rational approach for improving neoepitope/HLA complex stability and half-life on the cell surface, relative to unstable self-epitope/HLA complexes, through optimizing the peptide backbone conformation in addition to anchor residue interactions. This could ultimately lead to the selection of more efficient neoantigens, consistently with previous studies showing that the ability of tumor antigens to induce T cell responses that prevent tumor relapse correlates with p/MHC stability (6, 48).

As not all cancer patients who harbor a tumor-specific mutation that results in a neoepitope have the same HLA haplotype, it would be extremely beneficial to expand the repertoire of HLA molecules that bind and present a given therapeutic target. While sequence-based tools available at the IEDB (20) can provide highly reliable predictions of epitope binding for a range of HLA alleles, structural details of the predicted epitope/HLA complex relevant for interactions with TCRs are not provided by such methods. Complementary methods have been used to model interactions within peptide/HLA complexes by leveraging high-resolution structural data available in the PDB. These approaches employ flexible peptide docking to construct sequence specificity profiles by exploring different peptide/HLA combinations (30–32). Here, we utilize a comparative modeling approach with a fixed-peptide backbone while allowing for side-chain flexibility within the HLA groove to screen a large pool of HLA alleles for binding to our *ALK*-derived nonamer and decamer neoantigens (Figure 5; Figure S8 in Supplementary Material). High-ranking HLA alleles according to *Rosetta*’s binding energy consistently demonstrate a low percentile rank using the epitope prediction method recommended by IEDB, which further suggests a high probability of forming a tight complex with the neoepitopes (Figure S10 in Supplementary Material). We subsequently test our binding predictions and show that both the nonamer and decamer peptides form a stable complex with the common HLA-A*01:01 *in vitro*, albeit with decreased stability compared with the HLA-B*15:01 bound complex (Figure 2D; Figures S8 and S9 in Supplementary Material). The accuracy of the *Rosetta* models is highlighted by a comparison to our decamer/HLA-A*01:01 X-ray structure, which shows a backbone RMSD of 1.1 Å (Table S5 and Figure S12 in Supplementary Material). Our fixed-backbone approach is further supported by the observation that the conformation of the peptide backbone is maintained among X-ray structures containing different, high affinity nonamer peptides bound to HLA-B*15:01 (Figure S4E in Supplementary Material). Moreover, comparison of the decamer peptide conformation when bound to HLA-B*15:01 versus HLA-A*01:01, two alleles that share 51% of groove residues according to a pairwise sequence alignment, shows only a modest change (1.6 Å backbone RMSD) (Table S5 in Supplementary Material). In stark contrast, we observe a significant conformational change between the nonamer and decamer peptides in their crystallographic complexes with the same HLA-B*15:01 allele (2.7 Å backbone RMSD). These results suggest that peptide length defines the backbone conformation through the conservation of anchor residue interactions within a fixed-size class I MHC groove. This feature of peptide binding allows us to confidently model patient-specific neoepitope/HLA interactions in a high-throughput manner, using a single crystal structure containing the same peptide as template. Finally, our approach allows us to predict surface features of neoepitope/HLA complexes available for interactions with TCR molecules, toward further evaluating their immunogenicity. Within the current scope of our method, *Rosetta* accounts for conformational plasticity within the MHC groove by allowing for side-chain rotamer and limited backbone flexibility. Thus, accurate modeling of epitope binding is achieved given the template contains an MHC groove that is accommodated for a fixed-peptide length (i.e., to model a nonamer epitope, a template X-ray structure for a nonamer/HLA complex should be used). However, our current protocol cannot account for large changes in the backbone of the groove, which may be required to model peptides of shorter or longer length (49). Future improvements in our structure-based prediction procedure that account for this may be achieved using *Rosetta*’s Comparative Modeling (*RosettaCM*) hybridize (50) or *RosettaRemodel* (51).

To screen for CD8+ T cells that could recognize the tumor neoantigens, we focused our analysis on lymphocyte samples from healthy donors. We identify populations of CD8+ T cells which recognize our two *ALK* neoepitopes in a highly specific manner and with minimum cross-reactivity between them (Figures S13A–C in Supplementary Material). We observe approximately half the frequency (0.012 and 0.017%) of reactive CD8+ T cells for the HLA-B*15:01/nonamer tetramers relative to the frequency (0.024 and 0.028%) observed for HLA-B*15:01/decamer tetramers (Figure S13C in Supplementary Material), which may suggest differences in T cell recognition between the two epitopes. In addition, the percentage of reactive T cells against our two neoepitopes is comparable to values observed for the immunodominant SARS epitope (Figure S13C in Supplementary Material). While the nominal frequency of T cells specific for most p/MHC molecules ranges from 0.00005 to 0.01% (52, 53), our observed values for HLA-B*15 tetramers are within the range of specific T cells identified in previous reports of PBMC staining of healthy donors using HLA-B*15 tetramers (54). Our staining results support the recognition of our putative nonamer and decamer neoepitopes by CD8+ T cells, potentiating the ability for the epitopes to drive specific immune responses. Engagement of TCR molecules and triggering of signaling of CD8+ T cells are driven by interactions between the TCR complementarity-determining regions and specific peptide/HLA structural motifs (44, 55). Our detailed structural characterization provides further insight in the unique features that give rise to very distinct interface chemistries displayed by the two neoepitopes. It is likely the interplay between HLA complex stability and peptide surface features guides the engagement of CD8+ pools by the two neoantigens. Future studies in our group aim to identify the TCR(s) that can recognize our HLA displayed *ALK* neoepitopes toward the goal of characterizing the interface of the p/MHC–TCR complexes. Structural characterization of *ALK* p/MHC–TCR complexes will allow us to understand how the conformational plasticity observed in our nonamer and decamer neoepitopes dictates CD8+ T cell recognition (56) toward fostering the development of p/MHC–TCR complexes with improved stability in the immunological synapse (57).

In summary, we outline a novel approach toward robust, high-throughput identification and detailed characterization of highly stable putative neoantigen/HLA targets with desired T cell recognition features for cancer immunotherapy. Recently established technologies have enabled high-throughput, parallel detection of T cell specificities for a wide spectrum of epitopes through the combinatorial encoding of p/MHC multimers (57). Such methods have already been applied to monitor the prevalence of T cells that are reactive for established tumor epitopes (58). In addition, vaccination of cancer patients that display neoantigens can elicit a broad T cell response, both in terms of specificity and clonal diversity (59–61). The success of future cancer immunotherapies based on these technologies will depend on the ability to fine-tune the desired T cell responses toward specific tumor epitopes. Our data suggest that malleable structural features of the target neoepitope/MHC complex can be harnessed to achieve such a fine-tuning. Thus, our characterization of recurring, T cell-reactive neoepitopes together with their HLA specificities and molecular determinants of stability provide new screening tools and therapeutic targets to enable the development of personalized immunotherapies against NBL tumors.

## Materials and Methods

### NBL Sample Data Collection and ProTECT Analysis

One hundred NBL sequencing trios (normal and tumor DNA-seq, and tumor RNA-seq) were downloaded from the National Cancer Institute Genomics Data Commons (NCI-GDC) using the GDC Data Transfer Tool. Samples were all downloaded in BAM format and then converted back to the native paired FASTQ format using the Picard SamToFastq module. Some of the RNA-seq BAM files had reads in the pair mapped with separate read groups. These files were converted to FASTQ using an in-house python script.^1^ We processed the samples from raw FASTQ trios to neoepitopes prediction at a rate of ~6 h/sample on four Microsoft Azure machines (Supplementary Data S1 in Supplementary Material). MHC haplotypes for MHC class I and MHC class II are called from the sequencing data using PHLAT (62). The haplotype for a sample is decided based on a consensus decision of the three input haplotypes. Somatic point mutations were called using a panel of five mutation callers, MuTECT (63), MuSE (64), RADIA (65), SomaticSniper (66), and Strelka (67). Since most mutation callers are DNA centric, we allow mutations rejected by up to two of the callers through this first filter. The vcf of first-pass mutants is subjected to SNPEff (68) using indexes generated from the GENCODE v19 annotations for GRCh37 (69). The accepted mutations are further filtered more stringently using an in-house tool, Transgene,^2^ before being translated into mutant peptides. Library construction for sequencing can induce artificial oxidation of guanine bases (OxoG) (70) caused by high-energy sonication. These OxoG bases pair with thymine during PCR instead of their regular pairing partner, cytosine. This results in low allele fraction G>T or C>A substitutions seen predominantly in read 1 or read 2, respectively, in the FASTQ. Transgene filters variants arising solely form read 1 or read 2 in the alignment, and low allele-fraction mutants (<0.1 allele fraction) with no RNA-seq coverage. Since non-expressed proteins will never be picked up by the adaptive immune system, we filter events having low RNA-seq coverage. A mutation is filtered if the position has no evidence in the RNA (unexpressed ALT allele), there are reads spanning across, but none covering the position (splice variant), or if the gene is unexpressed. Filtered mutants are translated into peptides of length 2*n* − 1 for *n* = (9, 10, 15) using the GENCODE protein coding translations corresponding to the annotation used. Transcript-specific peptides are generated to account for known splice variants. The peptides generated by transgene are tested for binding against the inferred HLA haplotypes using the IEDB suite of MHC-I and MHC-II epitope predictors.^3^ Each 2*n* − 1-mer input peptide yields n calls for each allele in the HLA haplotype, for each *n* = (9, 10) for MHCI and *n* = 15 for MHC-II. Each call represents a combined consensus percent score of the peptide from a number of IEDB algorithms that have been trained on that MHC allele. These methods include an artificial neural network, a stabilized matrix method, a method that uses binding motif obtained from Combinatorial libraries, etc., and each method returns the percent rank of the input peptide:MHC combination versus a background set generated by the IEDB. The consensus score for a call is the median of the scores across all methods for that call. Peptides having a consensus percent score of greater than 5% (i.e., binders worse than the top 5% of the background set) are filtered as non-binders. Peptides having a consensus percent rank of greater than 5% (i.e., binders worse than the top 5% of a background set) are filtered as non-binders. The rank of the self-peptide for each filtered mutant is calculated using the same method. Peptides are grouped by the mutation and transcript(s) of origin into ImmunoActive Regions (IARs), i.e., regions likely to produce a peptide that will stimulate the immune system. IARs are ranked based on the affinity of the best contained binder, expression of the transcript(s) of origin, the promiscuity of the region (the predicted number of MHCs stimulated by peptides in the IAR), and the number of 10-mers in the IAR overlapping a 9-mer that binds to the same MHC as the 10-mer, with similar affinity. In the initial pilot, RNA-seq BAMs from six primary:relapsed pairs of samples were downloaded from the GDC and run through a reduced version of the pipeline using VCF files generated from the supplementary data from Eleveld et al. (16) containing predicted mutations. MHC haplotypes for these samples were decided based on the consensus calls from the primary and relapsed RNA-seq. All samples were run through version 2.3.2 of the ProTECT pipeline (freely available Docker version at https://quay.io/repository/ucsc_cgl/protect) on Microsoft Azure standard_G5 (32 CPUs, 448GB RAM, 6TB disk) or standard_D15_v2 (20 CPUS, 140GB RAM, 1TB disk).

### Recombinant Protein Expression and Purification

HLA-B*15:01 and HLA-A*01:01 genes containing a BirA tag were cloned into pET24+ plasmids and provided to us by the NIH Tetramer Core facility. For all *in vitro* experiments and the preparation of purified molecules for X-ray crystallography, we used soluble versions of the MHC heavy chain that lacks the BirA tag. Site directed mutagenesis to remove the BirA tag was performed using a QuikChange Lightning Multi-Site Kit (Agilent #210515) following the manufacturer’s instructions. Resulting DNAs encoding HLA-B*15:01 (heavy chain), HLA-A*01:01 (heavy chain), and human β_2_M (light chain) were transformed into *E. coli* BL21-DE3 (Novagen), expressed as inclusion bodies, and refolded using previously described methods (22). Briefly, *E. coli* growths with autoinduction (71) were pelleted by centrifugation and resuspended with 25 mL BugBuster (MilliporeSigma #70584) per liters of culture. Cell lysate was sonicated and subsequently pelleted by centrifugation (5,180 × *g* for 20 min at 4°C) to collect inclusion bodies. Inclusion bodies were resuspended with 25 mL of wash buffer (100 mM Tris pH 8, 2 mM EDTA, and 0.01% v/v deoxycholate), sonicated, and centrifuged again. Inclusion bodies were further resuspended in 25 mL of TE buffer (100 mM Tris pH 8, 2 mM EDTA) sonicated, and centrifuged. Following this, inclusion bodies are solubilized with 11 mL of resuspension buffer (100 mM Tris pH 8, 2 mM EDTA, 0.1 mM DTT, and 6 M guanidine–HCl). Solubilized inclusion bodies of heavy chain and light chain were mixed in a 1:3 M ratio and then added dropwise over 2 days to 1 L of refolding buffer (100 mM Tris pH 8, 2 mM EDTA, 0.4 M arginine HCl, 4.9 mM l-glutathione reduced, and 0.57 mM l-glutathione oxidized) containing 10 mg of synthetic peptide (Biopeptik). Refolding was performed for 4 days at 4°C without stirring then the sample was exhaustively dialyzed into SEC buffer (25 mM Tris pH 8 and 150 mM NaCl). Following this, the sample was concentrated with Labscale TFF system to 100 mL and further concentrated to a final volume of 5 mL using an Amicon Ultra-15 Centrifugal 10 kDa cutoff Filter Unit (Millapore Sigma). Purification was performed using SEC on a HiLoad 16/600 Superdex 75 pg with running buffer of 25 mM Tris pH 8 and 150 mM NaCl, followed by anion exchange chromatography using a mono Q 5/50 GL column and a 0–100% gradient of buffer A (25 mM Tris pH 8 and 50 mM NaCl) and buffer B (25 mM Tris pH 8 and 1 M NaCl). Finally, the purified protein was exhaustively buffer exchanged into 20 mM sodium phosphate pH 7.2 and 50 mM NaCl. The final sample was validated using LC–MS on an LTQ-Orbitrap Velos Pro MS instrument to confirm the presence of bound peptide.

### MHC Tetramerization

HLA-B*15:01 containing BirA tag was refolded together with either synthetically produced AQDIYRASY or AQDIYRASYY peptide (Biopeptik) and purified following methods described earlier. Purified protein was concentrated to 0.5 mg/mL and 500 µg was biotinylated using a BirA biotin-protein ligase bulk reaction kit (Avidity Cat no. bulk BirA) following the manufacturer’s instructions. An SDS-PAGE gel shift assay was performed to confirm the efficiency of the biotinylation reaction according to previously published protocols (72). The biotinylated protein sample was concentrated to 200 µL and split into two approximately 200 µg aliquots. For streptavidin–PE tetramers, 31.8 µL of 1 mg/mL of streptavidin-R-phycoerythrin (Prozyme cat no. PJRS25) was added 10 times in intervals of 10 min. For streptavidin–APC tetramers, 17.1 µL of 1 mg/mL streptavidin–allophycocyanin (Prozyme cat no. PJ27S) was added 10 times in intervals of 10 min. The final tetramer samples were stored at 4°C.

### Protein Crystallization

Purified HLA-B*15:01/AQDIYRASY, HLA-B*15:01/AQDIYRASYY, and HLA-A*01:01/AQDIYRASYY complexes lacking a BirA biotinylation tag were used for crystallization. Proteins were concentrated to 10–12 mg/mL in 50 mM NaCl, 25 mM Tris pH 8.0, and crystal trays were set up using 1:1 protein-to-buffer ratio at room temperature. For HLA-B*15:01/AQDIYRASY, small crystals appeared in initial screening using molecular dimensions JCSG-plus screen after 3 days in 100 mM HEPES pH 6.5 and 20% PEG 6000 and they were further optimized. Diffraction quality crystals were harvested and incubated from above conditions plus Al’s oil as a cryoprotectant and flash-frozen in liquid nitrogen before data collection. Diamond shaped diffraction quality crystals of HLA-B15:01/AQDIYRASYY were grown in crystallization buffer containing 100 mM HEPES, 2 M ammonium sulfate, and 2–4% PEG 400. Diffraction quality crystals of HLA-A*01:01/AQDIYRASYY were grown in 0.18 M magnesium chloride, 0.09 M sodium HEPES pH 7.5, 27% (v/v) PEG400, and 10% (v/v) glycerol. Crystals were flash-frozen in liquid nitrogen in a buffer containing the crystallization condition supplemented with 25% glycerol. All crystals used in this study were grown using the hanging drop vapor diffusion method. Data were collected from single crystals under cryogenic condition at Advanced Light Source (beam lines 8.3.1 and 5.0.1). Diffraction images were indexed, integrated, and scaled using Mosflm and Scala in the CCP4 package (73). Structures were determined by Phaser (74) using a previous structure of HLA-B*15:01 (PDB ID 1XR8) (25) and HLA-A*01:01 (PDB ID 1W72) (75) as search models. Model building and refinement were performed using COOT (76) and Phenix (77), respectively.

### Differential Scanning Fluorimetry

All DSF experiments were performed using an Applied Biosystems ViiA qPCR machine with excitation and emission wavelengths at 470 nm and 569 nm respectively, according to previously described protocols (23). Each sample was run in triplicates of 50 μL total volume using a 96 well-plate format. Proteins were buffer exchanged into the assay buffer which was 20 mM sodium phosphate at pH 7.2 and 50 mM NaCl. Individual wells contained a final concentration of 7 µM of the respective proteins and 10× SYPRO orange dye (ThermoFisher). To determine thermal stability of each sample, the temperature incrementally increased at a scan rate of 1°C/min from 25 to 95°C. Data analysis was performed using GraphPad Prism. Melting temperatures (*T*_m_) were determined by fitting the melting curves to a Boltzmann sigmoidal fit.

### Modeling MHC Molecules and Extracting Peptide/MHC-Binding Energies

The solved X-ray structure of HLA-B*15:01/AQDIYRASY complex was used to generate a structural model for HLA-B*15:01/ARDIYRASY using single-point mutagenesis in Pymol (The PyMOL Molecular Graphics System, Version 1.8 Schrödinger, LLC.) All Dunbrack rotamers (78) for the Arg2 side chain were considered manually, and the rotamer giving the lowest strain was used in our final structural model in Figure S6A in Supplementary Material. Peptide/MHC-binding energies were computed using the *Rosetta* software suite.^4^ Average binding energies of residue-specific interactions were calculated using the residue_energy_breakdown protocol in *Rosetta*.

To assess the ability of our *ALK* neoepitopes to bind to other HLA alleles, we performed homology-based structure simulations and computed p/MHC-binding energies *in silico*. An outline of our method is presented in Figure 4. Three-dimensional structural modeling and computation of p/MHC-binding affinities were performed using the *Rosetta* software suite (see text footnote 4). To carry out the modeling of homologous HLA alleles, we used *RosettaCM* protocol (50). The process of modeling high-resolution protein structures using *RosettaCM* primarily requires, that is, the sequence of the homolog is aligned with the sequence of a related known structure. It is subsequently followed by the generation of predicted 3D structures using restraints guided by a Monte Carlo sampling strategy. After performing the structure simulations of HLA alleles using our HLA-B*15:01 X-ray structure as a template, we carried out local refinement of the peptide and the MHC-binding groove. We kept backbone atoms fixed while allowing for conformational freedom of side-chain residues. The MHC-binding groove was defined by the HLA residues that were within 3.5 Å of the peptide. Local structure refinement allowed minimization of steric clashes introduced by the *RosettaCM* protocol. In addition, we refined only the peptide and the MHC-binding groove of the models to avoid noise that the full-atom refinement might introduce while trying to minimize the energy landscape at other regions and hence, making it difficult to extract accurate p/MHC-binding energies. At the local refinement stage, we generated a pool of refined structures from which we sampled low binding energy (or high-binding affinity) structures. Average binding energy was evaluated using the *Rosetta* energy function *talaris2014* (79, 80). The computation of binding energies was performed in the following steps: (1) we trimmed the MHC PDB file to remove the β_2_m and α3 domains, such that only the α1/α2 domains that form the peptide-binding groove were retained. (2) We performed local refinement of the MHC-binding groove and the peptide using *Rosetta*’s relax protocol (81), which allows the region of focus to be in the local optimum of the *Rosetta* force field. Using the relax protocol, we obtained a pool of 100 locally refined models. (3) We computed the binding energies of the relaxed models using the *InterfaceAnalyzer* protocol (79, 82) by separating the MHC and the peptide energy contributions and subtracting them from the energy of the bound p/MHC (30). (4) We then selected the lowest 10 binding energy models and report their average binding energies. The sequence identity score was computed using the BLOSUM62 matrix (33) because most of the HLA alleles (68%) showed up to 62% sequence similarity. To perform the simulation, we obtained the HLA sequences from European Bioinformatics Institute’s IPD-IMGT/HLA Database (29). We used ClustalOmega (83) to perform multiple sequence alignment of the HLA alleles before converting the alignment to *Rosetta*’s internal alignment format for homology modeling. Kullback–Leibler sequence logos were generated as previously described (84). Rosetta simulations were performed at the UCSC Baker cluster using 13 compute nodes with 32 cores per compute node (AMD Opteron(tm), 2.4 GHz Processor 6378). The total time used to model 2,904 HLA sequences was approximately 20,000 core hours.

### PBMC Staining

206 Cryopreserved PBMCs (CTL) from two healthy independent non-pooled HLA-B*15:01 donors were thawed and rested in phenol red free RPMI-1640 media supplemented with 10% FBS, 1% l-glut, and 1% Pen/Strep at 37°C for at least 1 h. Four independent PBMC staining experiments were run for each donor. 36 PBMCs were used for the nonamer/HLA-B*15:01, decamer/HLA-B*15:01, and SARS/HLA-B*15:01 double staining experiment. 11^6^ PBMCs were used for the decamer/HLA-B*15:01-APC and nonamer/HLA-B*15:01-PE experiment. After the resting period, cells were washed with 1× PBS, followed by staining with 4 µL of each tetramer, 5% CO_2_ for 10 min. An aqua amine-reactive dye (Invitrogen # L34957) was added for 10 min to assess cell viability, followed by the addition of an antibody cocktail (CD14, CD19, CD4, CD8) to stain for surface markers for an additional 20 min. The cells were washed with FACS buffer (PBS containing 0.1% sodium azide and 1% BSA) and sorted using an Aria C Flow Cytometer. Analysis of percentage of reactive CD8+ T cells was performed following gating on forward/side scattering for live lymphocytes (FSC+/SSC−), gating on Qdot− for live cells and gating on CD4−/CD8+ T cells.

## Data Availability

The refined coordinates and structure factors for the X-ray structures of HLA-B*15:01/AQDIYRASY, HLA-B*15:01/AQDIYRASYY, and HLA-A*01:01/AQDIYRASYY complexes have been deposited in the Protein Data Bank (www.rcsb.org) with PDB IDs 5TXS, 5VZ5, and 6AT9, respectively. The ProTECT pipeline is available for use under the Apache License v2.0 for academic users (https://github.com/BD2KGenomics/protect).

## Ethics Statement

All patients provided informed consent for analysis of health donor PBMCs according to specifications of the Children’s Hospital of Philadelphia.

## Author Contributions

SS, JM, DH, and NS conceptualized and designed the research. AR and AAM performed ProTECT analysis of sequencing data. SN and NS performed *Rosetta* comparative modeling simulations and binding energy calculations. JT, ACM, and KY prepared recombinant HLA samples and acquired LC–MS and DSF data. JT, ACM, and ST analyzed and interpreted X-ray crystallography data. MY, SN, ACM, and JT collected and analyzed PBMC tetramer staining data.

## Conflict of Interest Statement

The authors declare that the research was conducted in the absence of any commercial or financial relationships that could be construed as a potential conflict of interest.

## Footnotes

^1^https://github.com/arkal/random/process_rgs.py.

^2^https://github.com/arkal/Transgene.

^3^http://tools.iedb.org/mhci/.

^4^https://www.rosettacommons.org.
