# Supplementary material for: A Recurrent Mutation in Anaplastic Lymphoma Kinase with Distinct Neoepitope Conformations
Source: Front Immunol. 2018 Jan 30;9:99. doi: 10.3389/fimmu.2018.00099 (PMC5797543; doi:10.3389/fimmu.2018.00099)
Supplement: Supplementary file 1 [file Data_Sheet_1.pdf]

Supplementary Material

A Recurrent Mutation in Anaplastic Lymphoma Kinase with Distinct Neoepitope Conformations

Jugmohit S. Toor, Arjun A. Rao, Andrew C. McShan, Mark Yarmarkovich, Santrupti Nerli, Karissa Yamaguchi, Ada A. Madejska, Son Nguyen, Sarvind Tripathi, John M. Maris, Sofie R. Salama, David Haussler°, Nikolaos G. Sgourakis°

°To whom correspondence should be addressed: D.H. (haussler@soe.ucsc.edu) or N.G.S. (nsgourak@ucsc.edu)

Supplementary Data

Supplementary Data 1. The entire list of neoepitopes predicted from the six primary:relapsed TARGET NBL patients. Each sheet on the table describes the results from one sample including the mutated genes, the specific mutation, the neoepitopes and its corresponding binding MHC, and a binding score.

Supplementary Data 2. Summary of 100 TARGET NBL samples processed using ProTECT. Samples were processed in small groups across multiple Azure machines. Mutation rates predicted with and without OxoG filtering, and the corresponding CA/GT rates are provided for each sample. Samples in processing groups F1-15 denote samples that non-deterministically failed the automated runs. These samples were rerun individually to ensure successful completion.

Supplementary Data 3. Raw calls from the 100 TARGET NBL sample run.

Note: supplementary data will be provided at time of publication

Supplementary Tables

Protein:Mutation	Immuno Active Region	Samples with Expressed Mutation		Therapeutic MHC-I Allele (Frequency in Population)	Counts in COSMIC
		Primary	Relapse		
ALK:R1275Q*	AKIGDFGMA**[R/Q]**DIYRASYYR	5	1	HLA-B*15:01 (0.06)<br>HLA-A*02:01 (0.20)	101
ALK:F1174L	LMEALIISK**[F/L]**NHQNIVRCI	3	0	HLA-B*44:03 (0.02)	144
ALK:F1174I	LMEALIISK**[F/I]**NHQNIVRCI	1	0	HLA-B*13:02 (0.01)	7
ZNF717:Q716E	NVENPFIRR**[Q/E]**IFRSIKVFT	3	0	HLA-B*07:02 (0.03)	0
KCNJ12:E378K	LPSANSFCY**[E/K]**NELAFLSRD	2	2	HLA-A*23:01 (0.01)	0
ITGB2:I712T	AIVGGTVAG**[I/T]**VLIGILLLV	2	0	HLA-A*02:01 (0.02)	0
USP6:R69W	TAREAKKIR**[R/W]**EMTRTSKWM	2	0	HLA-A*24:02 (0.38)<br>HLA-C*04:01 (0.15)	0
TSC22D1:T961K	LFPLKVLPL**[T/K]**TPLVDGEDE	2	0	HLA-C*03:03 (0.04)<br>HLA-C*04:01 (0.15)	0
NRAS:Q61K	LLDILDTAG**[Q/K]**EEYSAMRDQ	1	1	HLA-A*01:01 (0.04)	1287

Supplementary Table 1. ProTECT analysis of 100 primary and 6 primary:relapsed pairs of TARGET NBL samples. The first column shows identified proteins (in italics) with their respective mutation observed in tumors. The second column shows the ImmunoActive Region of each protein with the format [X /Y] in bold denoting wild-type and mutant amino acids, respectively. The third column shows the number of samples with the expressed mutation in primary and relapsed patients. The fourth column shows the best predicted therapeutic MHC allele, which is defined as either an allele shared by samples expressing the same mutation or an allele with the best predicted binding score. For each allele, the frequency (denoted in parenthesis) is in Caucasian populations. The fifth column shows counts in COSMIC that refer to the sum total of unique entries in the Catalog of Somatic Mutations in Cancer database (1) for each mutation.

	HLA-B*15:01/ AQDIYRASY	HLA-B*15:01/ AQDIYRASYY	HLA-A*01:01/ AQDIYRASYY
**Data collection**			
Beam line	ALS(8.3.1)	ALS(5.0.1)	ALS(8.3.1)
Space group	$P2_12_12_1$	$P6_122$	$P32_221$
Unit cell dimensions (a,b,c) (Å), (α, β, γ (º)	50.44, 82.19, 109.85 90, 90, 90	112.89, 112.89, 177.38 90, 90, 120	81.41, 81.41, 118.281, 90, 90, 120
Resolution Range (Å) (highest shell)*	45.84 - 1.697 (1.758 - 1.697)	97.77-2.59 (2.71-2.59)	45.31-2.95 (3.13-2.95)
Wavelength (Å)	1.00	1.00	1.12
Total observations	448746 (43022)	206944 (24966)	82897 (12531)
Unique reflections	51238 (5026)	21507 (2547)	9982 (1590)
Completeness (%)	99.93 (99.33)	100 (100)	99.8 (99.4)
R_{merge}	9.6 (74.4)	10.6 (135.2)	9.4 (77.1)
<I/σ>	11.22 (1.83)	14.6 (1.9)	16.2 (3.0)
CC1/2	0.998 (0.812)	0.998 (0.778)	0.998 (0.876)
Redundancy (highest shell)	8.8 (8.6)	9.6 (9.8)	8.3 (7.9)
**Refinement**			
R_{work} %/ R_{free} %	15.7(19.5)	21.6(26.5)	22.4(30.7)
Number of non-hydrogen atoms	3811	3275	3153
Protein	3267	3184	3153
Ligand/ion	-	67	-
Water	544	24	-
B-factor (Wilson)	25.8	65.8	78.18
RMSD Bond length (Å)	0.012	0.008	0.003
RMSD Bond angle	1.34	1.01	0.720
Ramachandran favored(%) /Ramachandran outliers (%)	97.6/0.0	94.2/0.0	92/0.2

¶¶ $R_{work} = ||F_{obs}(h)|-|F_{calc}(h)|| / {}_{h}|F_{obs}(h)|$, where $F_{obs}(h)$ and $F_{calc}(h)$ are the observed and calculated structure factors, respectively.

‡ R_{free} is the R value obtained for a test set of reflections consisting of a randomly selected 5% subset of the data set excluded from refinement.

Supplementary Table 2. X-ray data collection and structure refinement statistics for the neoepitope/HLA structures. Data was collected on single crystals. Values in parentheses are for the highest resolution shell.

AQDIYRASY	HLA-B*15:01	Distance (Å)	AQDIYRASYY	HLA-B*15:01	Distance (Å)
Ala1-N	Tyr7-OH	2.9	Ala1-N	Tyr171-OH	2.8
Ala1-N	Tyr171-OH	2.6	Ala1-N	Tyr7-OH	2.9
Ala1-O	Tyr159-OH	2.6	Ala1-O	Tyr159-OH	2.6
Gln2-N	Glu63-Oε1	2.9	Gln2-N	Tyr7-OH	3.5
Gln2-N	Tyr7-OH	3.4	Gln2-N	Glu63-Oε2	2.7
Gln2-O	Arg62-Nη1	2.8	Gln2-Oε1	Glu63-O	3.3
Gln2-O	Arg62-Nη2	3.0	Gln2-Oε1	Glu67-Oγ	3.2
Gln2-Oε1	Glu63-O	3.3	Gln2-Oε1	Glu63-Oε2	3.4
Gln2-Oε1	Glu63-Oε1	3.0	Gln2-Nε2	Ser67-Oγ	3.1
Gln2-Nε2	Tyr9-OH	2.8	Gln2-Nε2	Tyr97-OH	3.2
Asp3-N	Tyr99-OH	3.1	Gln2-Nε2	Asn70-Nδ2	3.5
Asp3-N	Tyr159-OH	3.6	Asp3-N	Tyr159-OH	3.6
Arg6-Nε	Glu152-Oε1	3.5	Asp3-N	Tyr99-OH	3.1
Arg6-Nε	Glu152-Oε2	2.8	Arg6-O	Tyr74-OH	2.8
Arg6-NH2	Glu152-Oε1	3.2	Arg6-Nε	Asp114-Oδ2	2.3
Arg6-NH2	Trp156-Nε1	3.4	Arg6-NH1	Trp156-O	3.3
Ser8-O	Lys146-Nζ	3.5	Arg6-NH2	Asp114-Oδ2	3.0
Ser8-O	Trp147-Nε1	2.9	Ala7-O	Thr73-Oγ1	2.7
Ser8-Oγ	Glu76-Oε2	2.8	Tyr9-O	Trp147-Nε1	3.0
Ser8-Oγ	Lys146-Nζ	2.9	Tyr10-N	Ser77-Oγ	2.6
Tyr9-O	Asn80-Nδ2	2.8	Tyr10-O	Lys146-Nζ	3.5
Tyr9-O	Tyr84-OH	3.2	Tyr10-O	Thr146-Oγ1	2.8
Tyr9-O	Lys146-Nζ	2.9	Tyr10-O	Tyr84-OH	2.5
Tyr9-N	Ser77-Oγ	3.0	Tyr10-OH	Arg97-Nη1	3.2
Tyr9-OH	Arg97-Nη1	3.5	Tyr10-OH	Ser116-Oγ	2.9
Tyr9-OH	Ser116-Oγ	2.6			

Supplementary Table 3. Table detailing molecular interactions within 3.6 Å between residues of the nonamer (AQDIYRASY) and decamer (AQDIYRASYY) peptide and residues of the HLA-B*15:01 groove in our X-ray structures (Figure 3).

AQDIYRASYY	HLA-A*01:01	Distance (Å)
Ala1-N	Tyr7-OH	3.2
Ala1-N	Tyr171-OH	3.0
Ala1-O	Tyr159-OH	2.7
Gln2-N	Glu63-Oε1	2.5
Gln2-Oε1	Asn66-O	3.4
Asp3-N	Tyr99-OH	3.2
Asp3-O	Tyr5-N	2.9
Asp3-Oδ1	Arg156-Nη1	3.3
Asp3-Oδ2	Arg156-Nη1	2.8
Ile4-O	Arg6-N	3.1
Tyr5-N	Asp3-O	2.9
Tyr5-OH	Arg114-Nη2	2.6
Arg6-N	Arg156-Nη2	3.1
Arg6-N	Ile4-O	3.1
Arg6-O	Ser8-N	3.4
Arg6-Nη2	Gln155-Nε2	3.4
Ala7-N	Arg156-Nη2	3.0
Ser8-O	Arg6-O	3.4
Ser8-O	Asn77-Nδ2	3.4
Ser8-Oγ	Arg6-N	3.3
Tyr9-O	W147-Nε1	2.7
Tyr10-N	Asn77-Oδ1	2.6
Tyr10-O	Tyr84-OH	2.3
Tyr10-O	Thr-Oγ1	3.2
Tyr10-O	K146-Nζ	2.7
Tyr10-OH	Asp116-Oδ1	2.5

Supplementary Table 4. Table detailing molecular interactions within 3.6 Å between residues of the decamer (AQDIYRASYY) peptide and residues of the HLA-A*01:01 groove in our X-ray structure (Figure 3).

**Peptide/HLA**<br>***Structure method***		**Peptide**<br>**RMSD (Å)**
**AQDIYRASY/HLA-B*15:01**<br>***X-ray***	**AQDIYRASYY/HLA-B*15:01**<br>***X-ray***	**All atom**<br>**4.0**
		**Heavy atom**<br>**2.7**
**AQDIYRASYY/HLA-B*15:01**<br>***X-ray***	**AQDIYRASYY/HLA-A*01:01**<br>***X-ray***	**All atom**<br>**1.7**
		**Heavy atom**<br>**1.6**
**AQDIYRASYY/HLA-A*01:01**<br>***Rosetta Homology Model***	**AQDIYRASYY/HLA-A*01:01**<br>***X-ray***	**All atom**<br>**1.7**
		**Heavy atom**<br>**1.1**

Supplementary Table 5. Comparison of the structure of nonamer (AQDIYRASY) and decamer (AQDIYRASYY) ALK neoepitopes bound to HLA-B*15:01 or HLA-A*01:01 from our X-ray structures or from the *Rosetta* homology model. For the *Rosetta* homology model of AQDIYRASYY/HLA-A*01:01, the structural template was the X-ray structure AQDIYRASYY/HLA-B*15:01 as described in the main text. RMSD was determined in PyMOL. RMSD – root mean squared deviation. Heavy atoms – Cα , CO N and O.

Supplementary Figures

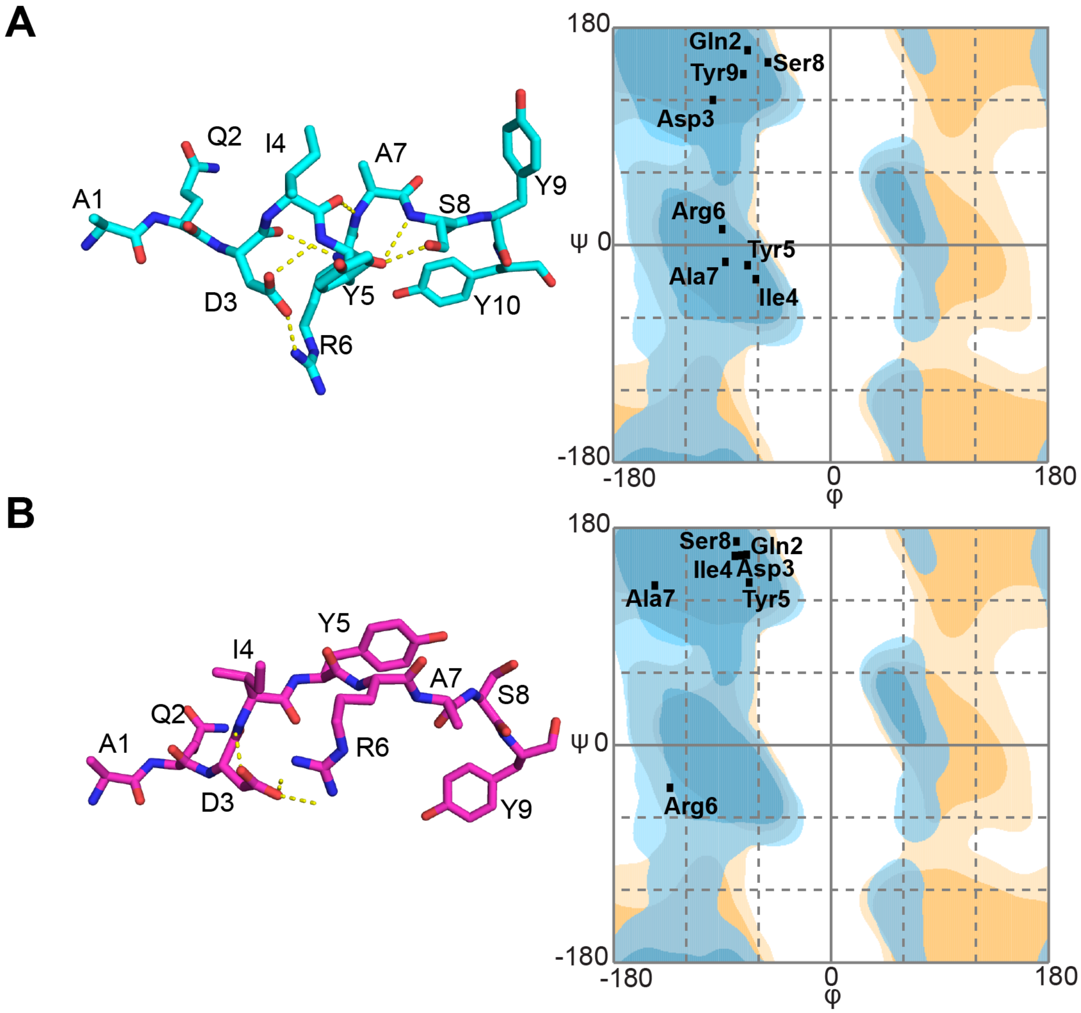

Supplementary Figure 1. **The decamer epitope forms a 3_{10} helix structure when bound to HLA-B*15:01. (A)** (Left) The decamer (cyan sticks) and **(B)** nonamer (magenta) peptides are shown with the hydrogen bonding network represented with yellow dashes. (Right) The Ramachandran plot showing the φ/ψ angles of each residue of the decamer (A) and nonamer (B) peptide in the binding groove of HLA-B*15:01. The φ/ψ were computed and plotted for each peptide residue using the RAMPAGE server (2). The diagram is divided into favored (dark blue), allowed (light blue), and disallowed (orange) regions.

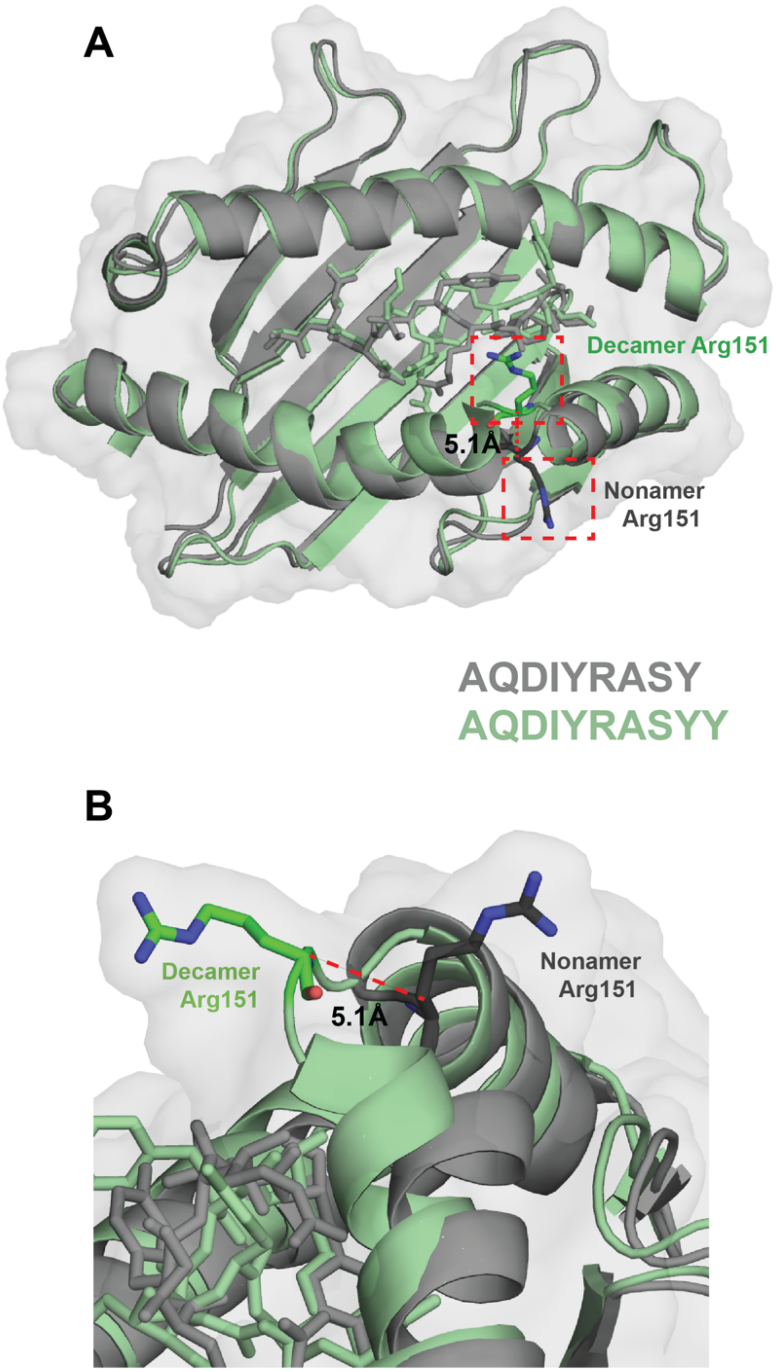

Supplementary Figure 2. Widening of the HLA-B*15:01 binding groove in the nonamer bound complex relative to the decamer bound complex. (A) and **(B)** show two angles of a superposition of the nonamer (gray) and decamer (green) bound HLA-B*15:01 X-ray structures from Figure 3. A widening of 5.1 Å in the α2 helix near Arg151 is observed (shown with red dotted line). The backbone heavy atom RMSD of the two structures is 0.815 Å. Notably, Arg151 of the decamer complex (dark green and blue sticks) is oriented inwards towards the binding groove, while Arg151 orients away from the binding groove in the nonamer complex (dark gray and blue sticks).

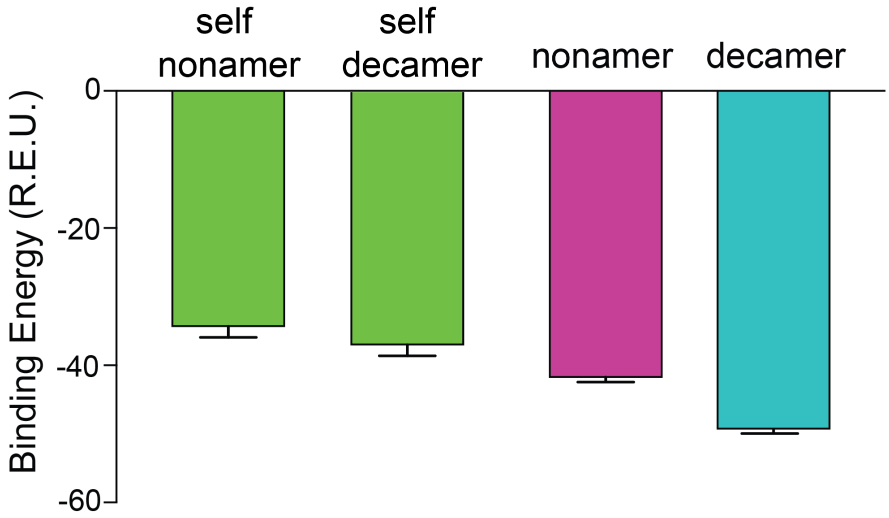

Supplementary Figure 3. Structure-based binding energy calculations of HLA-B*15:01 bound to the self, the nonamer, and the decamer epitopes. Binding energies in *Rosetta* energy units (R.E.U.) showing that the self-peptides have an unfavorable binding energy compared to either the nonamer or the decamer neoepitopes. This is supported by the loss of favorable interactions with groove residues observed in threaded models of the self-peptide using our HLA-B*15:01 X-ray structure as a template (Supplementary Figure 6A).

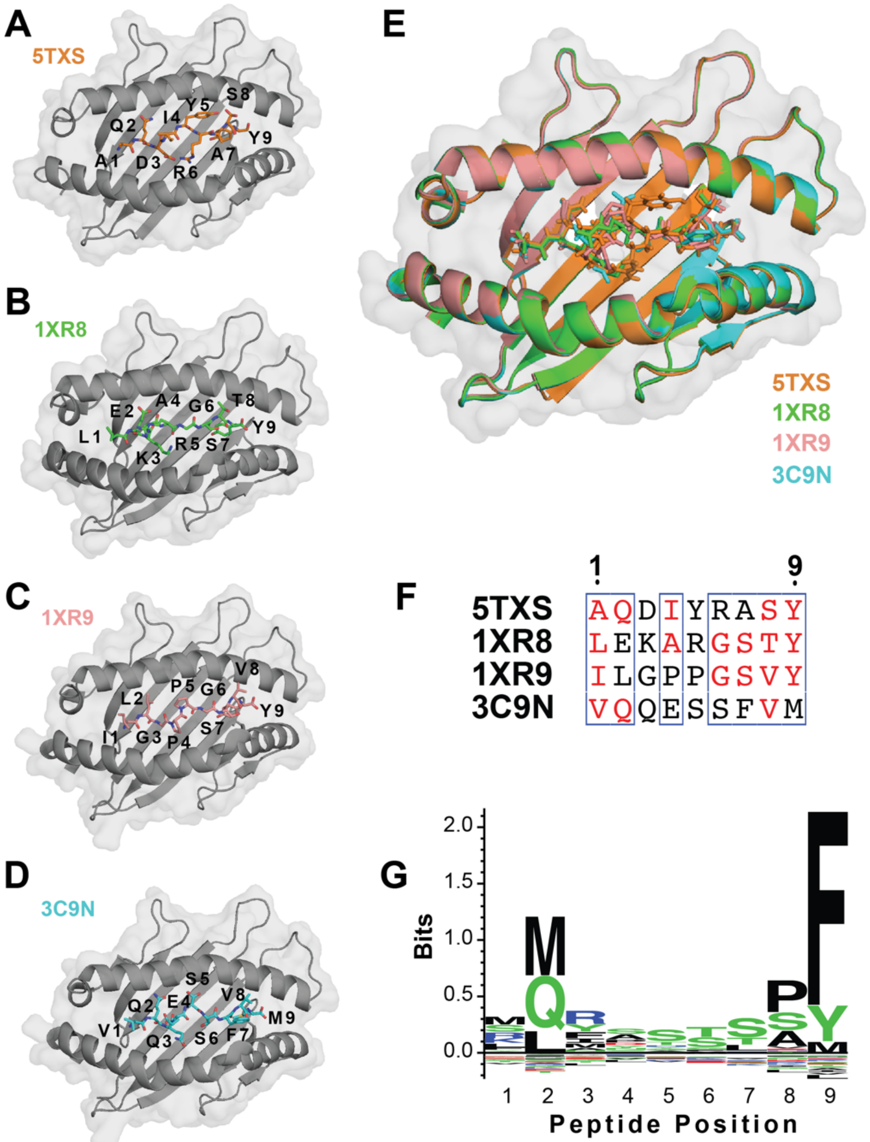

Supplementary Figure 4. Similar extended conformations observed for HLA-B*15:01 complexes with different nonamer peptides. X-ray structures showing the binding groove of HLA-B*15:01 complexed with **(A)** human *ALK* derived AQDIYRASY nonamer from this study (PDB 5TXS), **(B)** Epstein-Barr virus derived LEKARGSTY nonamer (PDB 1XR8), **(C)** human ubiquitin-conjugating enzyme-E2 derived ILGPPGSVY nonamer (PDB ID 1XR9), and **(D)** SARS coronavirus derived VQQESSFVM nonamer (PDB ID 3C9N). **(E)** An overlay of the binding groove of each HLA-B*15:01 X-ray structure showing backbone heavy atom RMSDs in the range of 0.5 to 1 Å. **(F)** Primary sequence alignment of each epitope sequence performed using ClustalOmega (3) and analyzed using ESPript 3 (4). **(G)** Kullback-Leibler sequence logo for the peptide motif (with respect to binding to HLA-B*15:01) showing preferred and tolerated amino acids for each position where position two and nine are the primary anchor positions. Quantitative peptide/HLA B*15:01 binding data obtained from the IEDB database were used to generate the sequence logo using Seq2Logo (5).

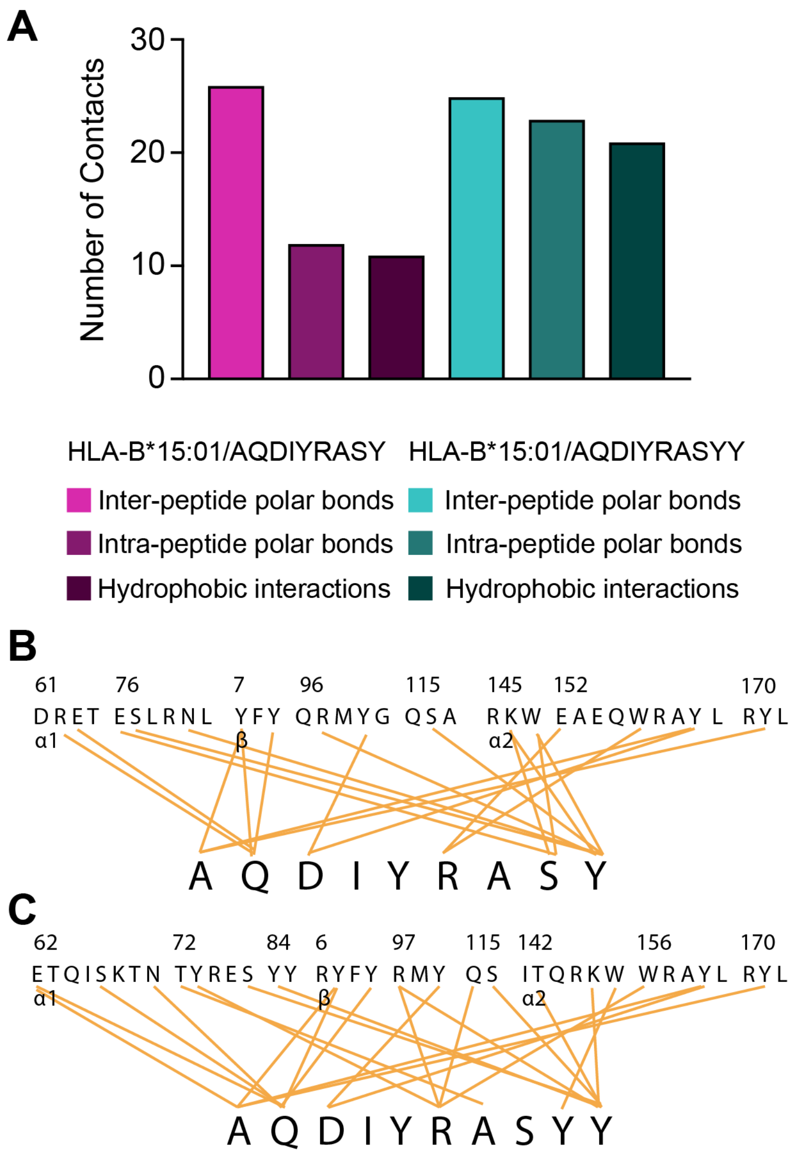

Supplementary Figure 5. Comparison of peptide-MHC interactions observed in the nonamer and decamer X-ray structures. (A) Quantitative comparison of different types of molecular contacts: peptide/MHC (inter-peptide polar), peptide/peptide (intra-peptide polar), peptide/MHC (hydrophobic) observed in our two peptide/HLA-B*15:01 X-ray structures shown in Figure 3. We used a distance cutoff of 3.6 Å between the nonamer (magenta) or decamer (teal) peptide and the binding groove. A detailed map of peptide residue interactions with different structural regions of the MHC groove (α_1-helix, β-sheet, α_2-helix) is further highlighted for the nonamer **(B)** or the decamer peptide structure **(C)**.

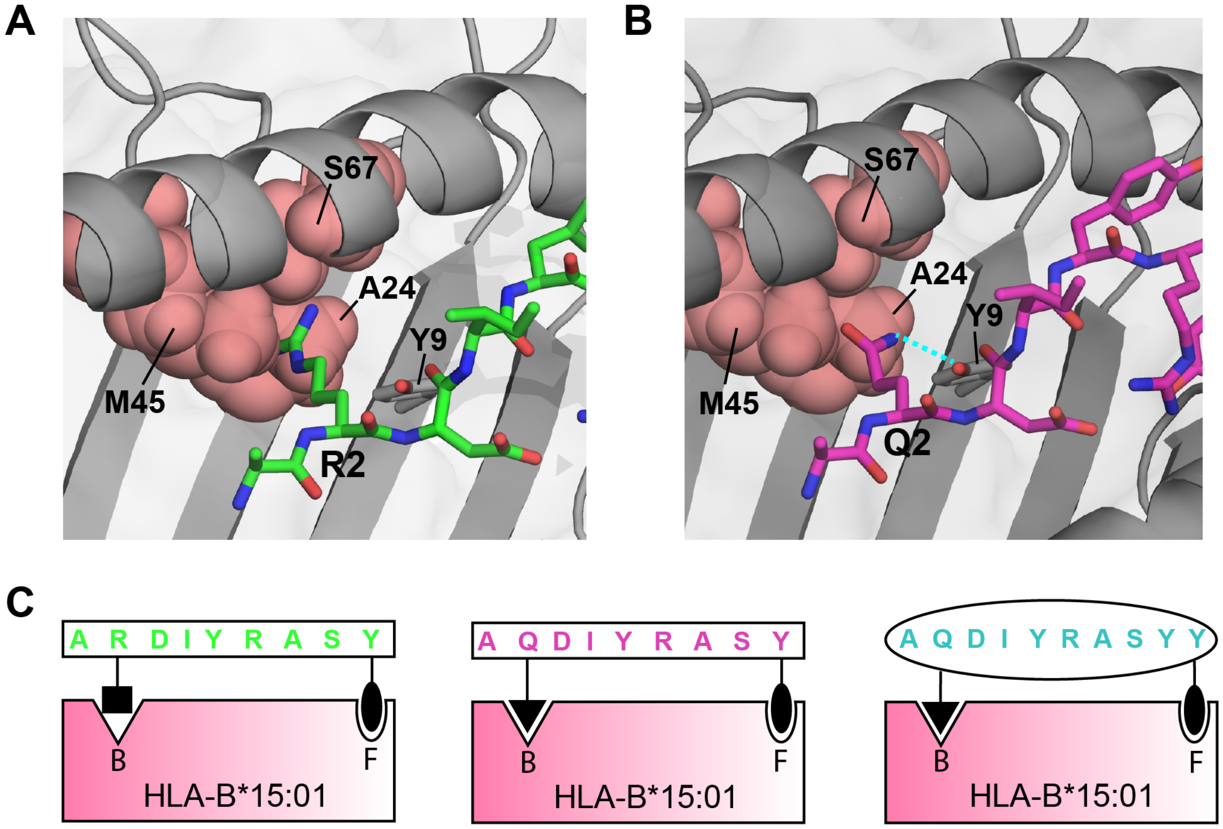

Supplementary Figure 6. **Structural modeling of the self-peptide complex with HLA-B*15:01.** **(A)** Structural model of HLA-B*15:01 bound to the self-peptide (ARDIYRASY, green sticks); the placement of the Arg2 sidechain results in unfavorable interactions with Ala24, Met45 and Ser67 of HLA-B*15:01 (light red spheres). **(B)** The X-ray structure of nonamer (AQDIYRASY, magenta sticks) bound to HLA-B*15:01, used as a modeling template for (A), highlights the smaller sidechain of Gln2 which is well-accommodated in the MHC groove. A hydrogen bond is further detected between Tyr9 of the HLA binding groove and Gln2 (cyan dotted line) in (B), but not with Arg2 in (A). **(C)** The models underlying self-peptide, nonamer peptide, and decamer peptide association with HLA-B*15:01 reinstates the fact that Arg2 of the self-peptide inhibits it from binding HLA-B*15:01, while the nonamer and decamer peptides both form high affinity complexes with distinct structural features displayed to T cells.

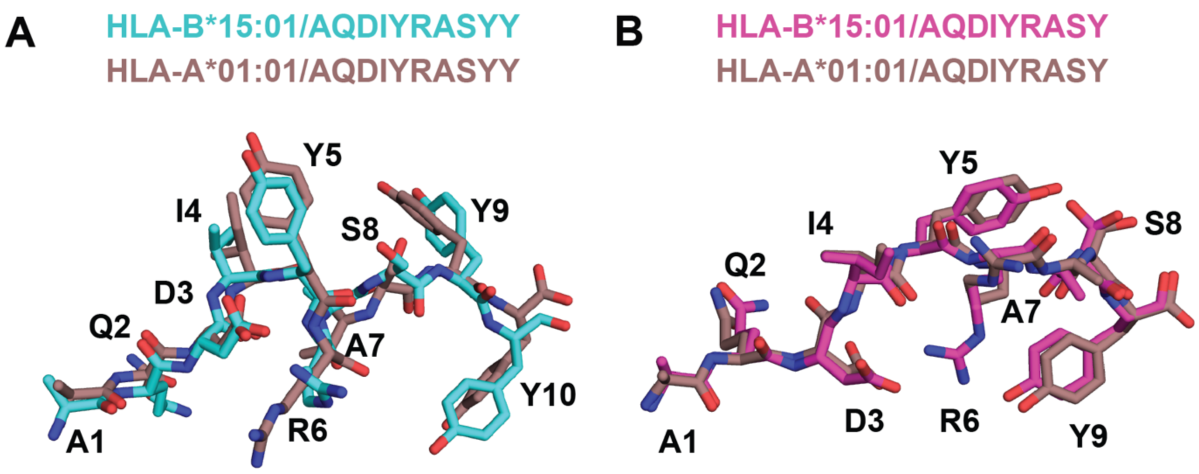

Supplementary Figure 7. Conformation of decamer and nonamer peptide displayed by HLA-A*01:01 from structural modeling. (A) 3_{10} helical conformation of the decamer peptide observed when bound to HLA-B*15:01 (cyan) in our X-ray structure or to HLA-A*01:01 (brown) in comparative modeling simulations. **(B)** Extended conformation of the nonamer peptide observed when bound to HLA-B*15:01 (green) in our X-ray structure or to HLA-A*01:01 (brown) in comparative modeling simulations.

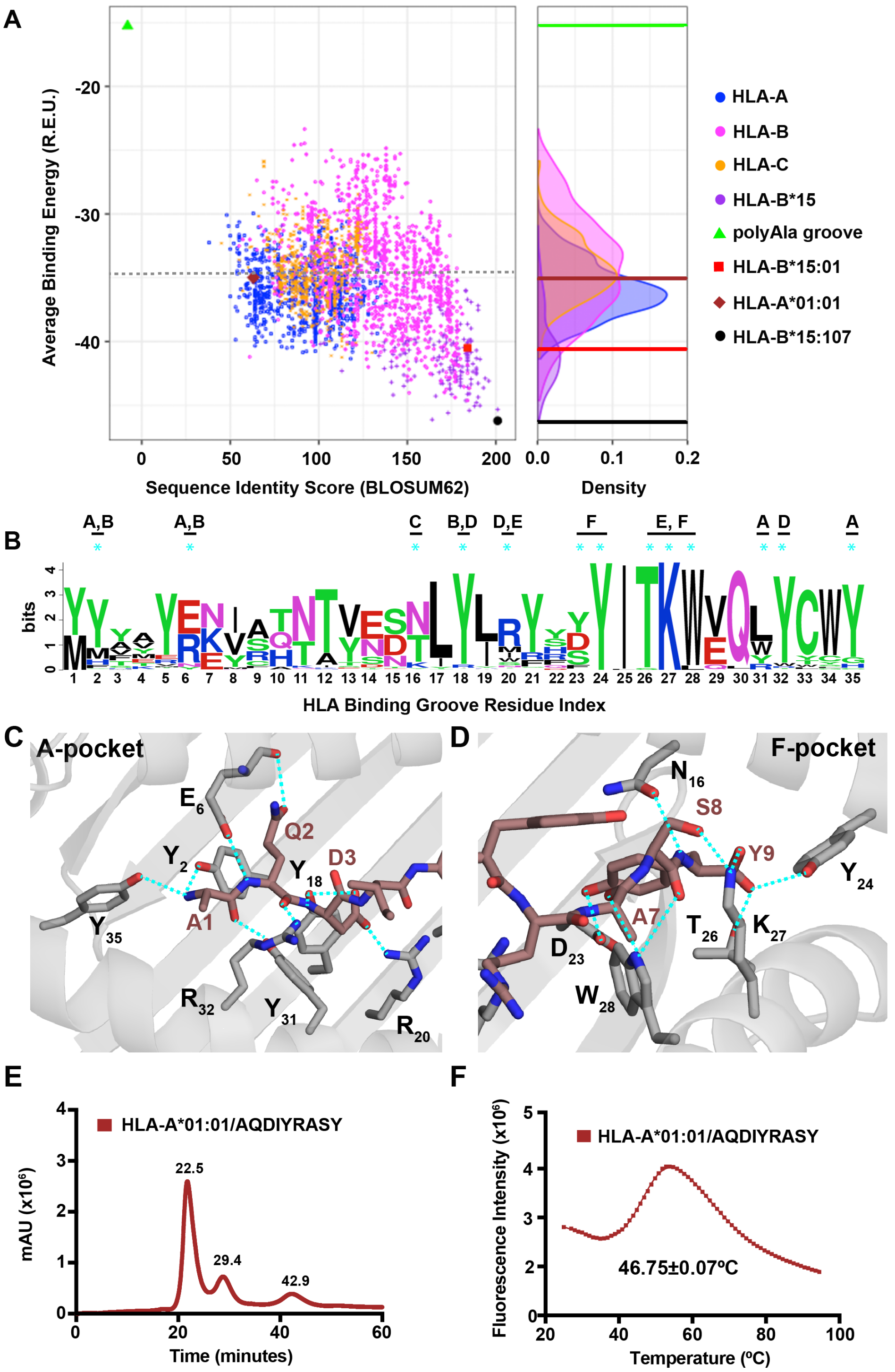

Supplementary Figure 8. Evaluating the HLA binding repertoire of *ALK* neoepitope nonamer AQDIYRASY using structure-based binding energy calculations. (A) *Rosetta* binding energies calculated from structure modeling of 2,904 unique HLA alleles from the IPD-IMGT/HLA Database (6), for the *ALK* neoepitope nonamer (AQDIYRASY) plotted as a function of sequence similarity to the top binding allele, HLA-B*15:107 (black circle). The binding energy of nonamer in our HLA-B*15:01 X-ray structure is shown as a reference (red square). A negative control was performed with a mock HLA allele where all residues in the binding groove were

replaced with Ala (polyAla groove, green triangle), which shows high binding energy. The corresponding distribution of the HLA alleles on the binding energy landscape is captured in the density plot shown on the right. Sequence identity scores were calculated using the BLOSUM62 (7) matrix**.** R.E.U.: *Rosetta* Energy Units **(B)** Kullback-Leibler sequence logo (5) derived from multiple sequence alignment using ClustalOmega (3) of peptide binding groove residues from all the HLA alleles that exhibit better binding energies than HLA-A*01:01 (brown diamond), indicated with a gray dotted line in (A). MHC residues with polar contacts to the peptide are denoted with a cyan asterisk with corresponding MHC pocket noted. **(C)** and **(D)** Threaded structural model of HLA-A*01:01 displaying nonamer peptide. Polar contacts between the MHC groove (gray sticks) and peptide (brown sticks) are shown with cyan dotted lines in the HLA-A*01:01 A-, B-, and D-pockets (C) or C-, E-, and F-pockets (D). The residue index for each interacting MHC residue is denoted with the corresponding number from (B) using subscripts. Peptide residues (non-indexed) are labeled without subscripts. **(E)** SEC trace of HLA-A*01:01 refolded with *ALK* derived nonamer peptide AQDIYRASYY with p/MHC complex eluting at 29.4 min, similar to the SEC elution of HLA-A*01:01 bound complex decamer (Supplementary Figure 8A). The presence of the nonamer peptide in the HLA-A*01:01 complex was confirmed by LC-MS. **(F)** DSF of HLA-A*01:01 refolded *in vitro* with *ALK* neoepitope nonamer (AQDIYRASY) has decreased thermal stability of 46.8°C relative to HLA-B15*01:01 refolded with the same peptide (Figure 2B, 59.3°C) and HLA-A*01:01 refolded with the decamer (47.9°C, Supplementary Figure 8B).

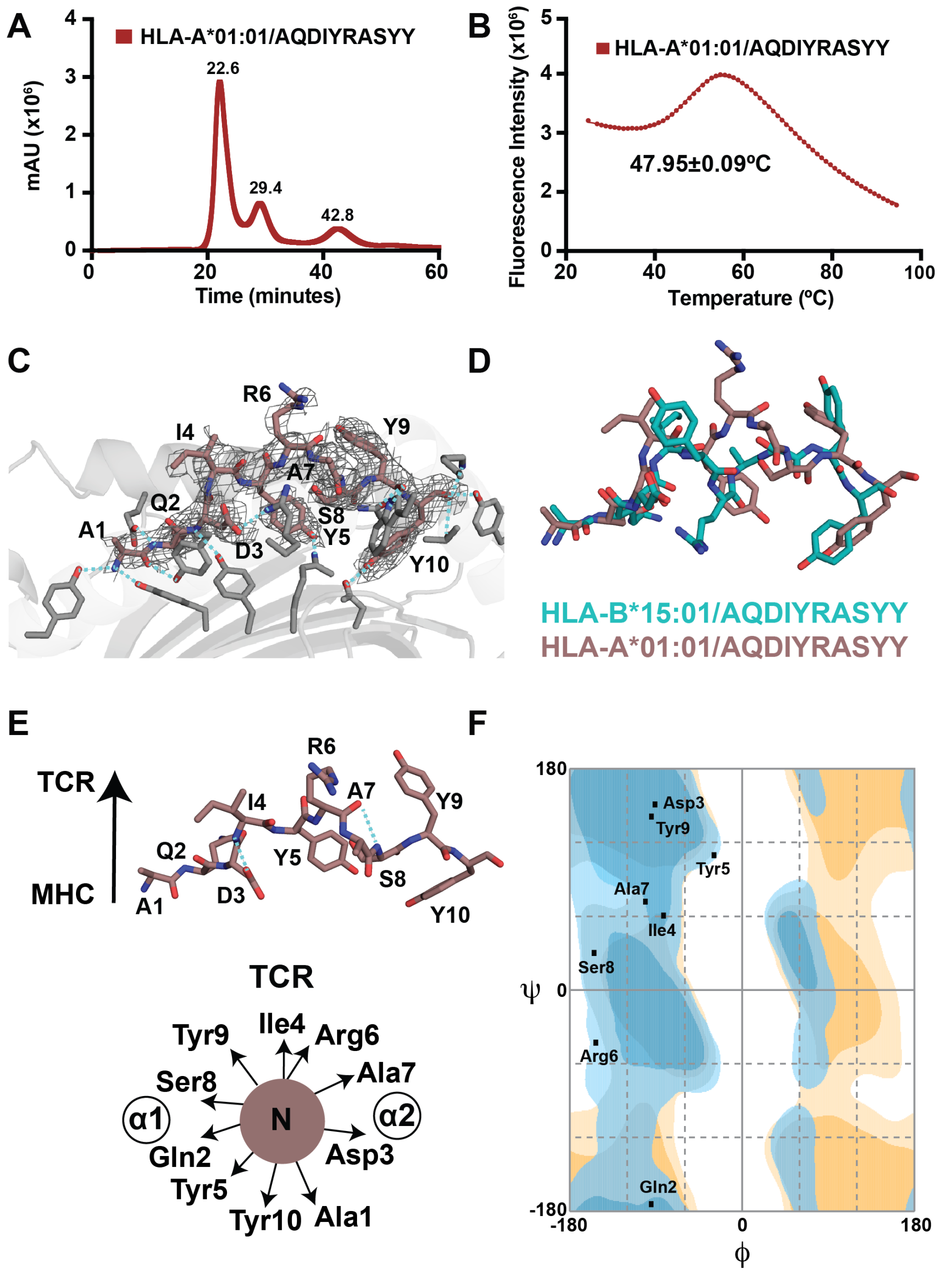

Supplementary Figure 9. Structural characterization of ALK neoepitope displayed by HLA-A*01:01. (A) SEC trace of HLA-A*01:01 refolded with *ALK* derived decamer peptide AQDIYRASYY with p/MHC complex eluting at 29.4 min, similar to the SEC elution of HLA-B*15:01 bound complex (Figure 2A). The presence of the decamer peptide in the HLA-A*01:01 complex was confirmed by LC-MS. **(B)** DSF of HLA-A*01:01 refolded *in vitro* with *ALK* neoepitope decamer (AQDIYRASYY) has decreased thermal stability of 47.9°C relative to HLA-B15*01:01 refolded with the same peptide (Figure 2B, 59.3°C). **(C)** Decamer peptide (brown sticks) with 2Fo-Fc electron density maps contoured at 1.6 σ within the groove of HLA-A*01:01. Cyan dashes represent polar contacts between the peptide and selected MHC residues (gray sticks). **(D)** Conformation of the decamer peptide observed when bound to HLA-B*15:01 (cyan) or HLA-A*01:01 (brown) in our X-ray structures. **(E)** (top) Structural features of AQDIYRASYY

displayed to T cells by HLA-A*01:01. (bottom) Sidechain orientation of the decamer peptide as viewed from the top axis of the peptide highlighting the placement of different residues. **(F)** Ramachandran plot showing the φ/ψ angles of each residue of the decamer peptide in the binding groove of HLA-A*01:01. The φ/ψ were computed and plotted for each peptide residue using the RAMPAGE server (2). The diagram is divided into favored (dark blue), allowed (light blue), and disallowed (orange) regions.

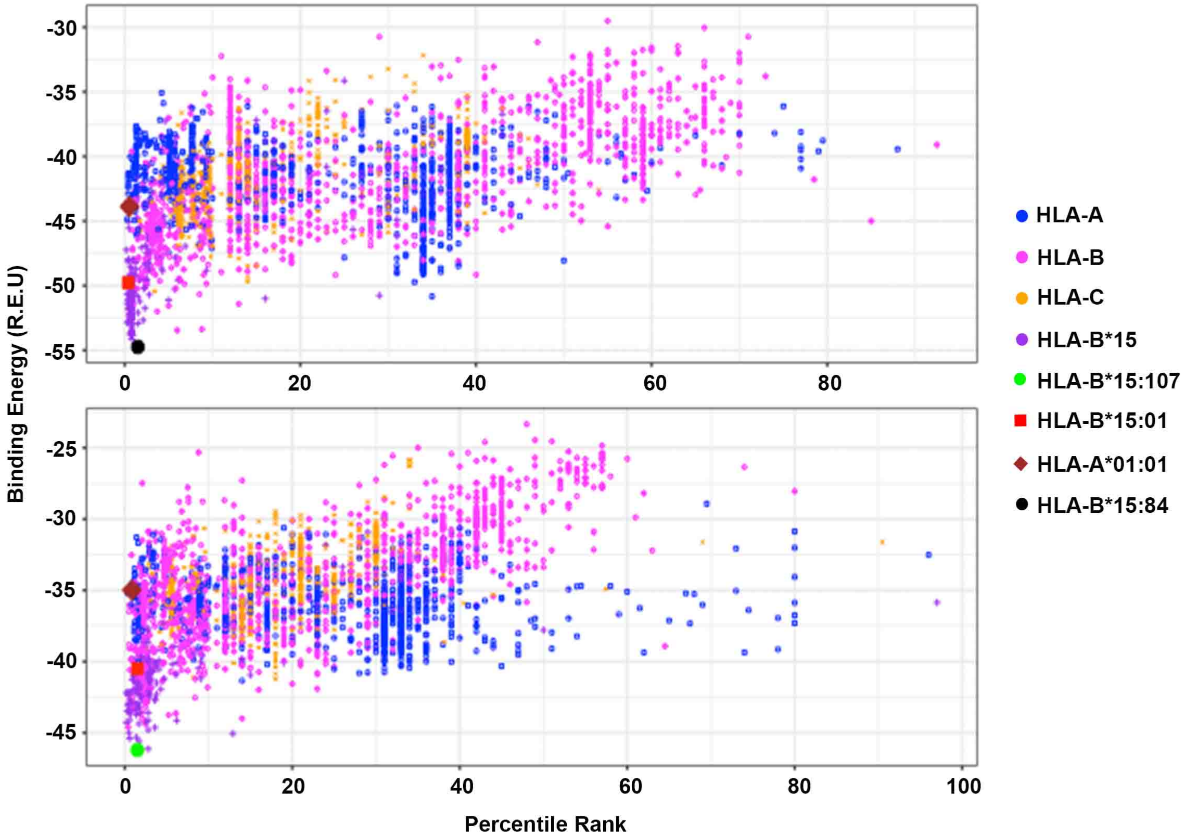

Supplementary Figure 10. Structure-based binding energies versus IEDB epitope predictions. Paired *Rosetta* binding energies and Immune Epitope Database (IEDB) (8) percentile ranks calculated for 2,904 unique HLA alleles from the IPD-IMGT/HLA Database (6). Results are shown for the *ALK* decamer (AQDIYRASYY) (top panel) and nonamer (AQDIYRASY) (bottom panel) neoepitopes. Percentile ranks were computed using the recommended prediction method by IEDB, i.e. either a consensus score derived from a series of prediction methods such as artificial neural networks (ANN), stabilized matrix method (SMM) and scoring matrices derived from combinatorial peptide libraries (CombLib) (9), or a single score from NetMHCpan (10). A low percentile rank indicates that the corresponding HLA allele is a good binder to the *ALK* neoepitope. R.E.U.: *Rosetta* Energy Units.

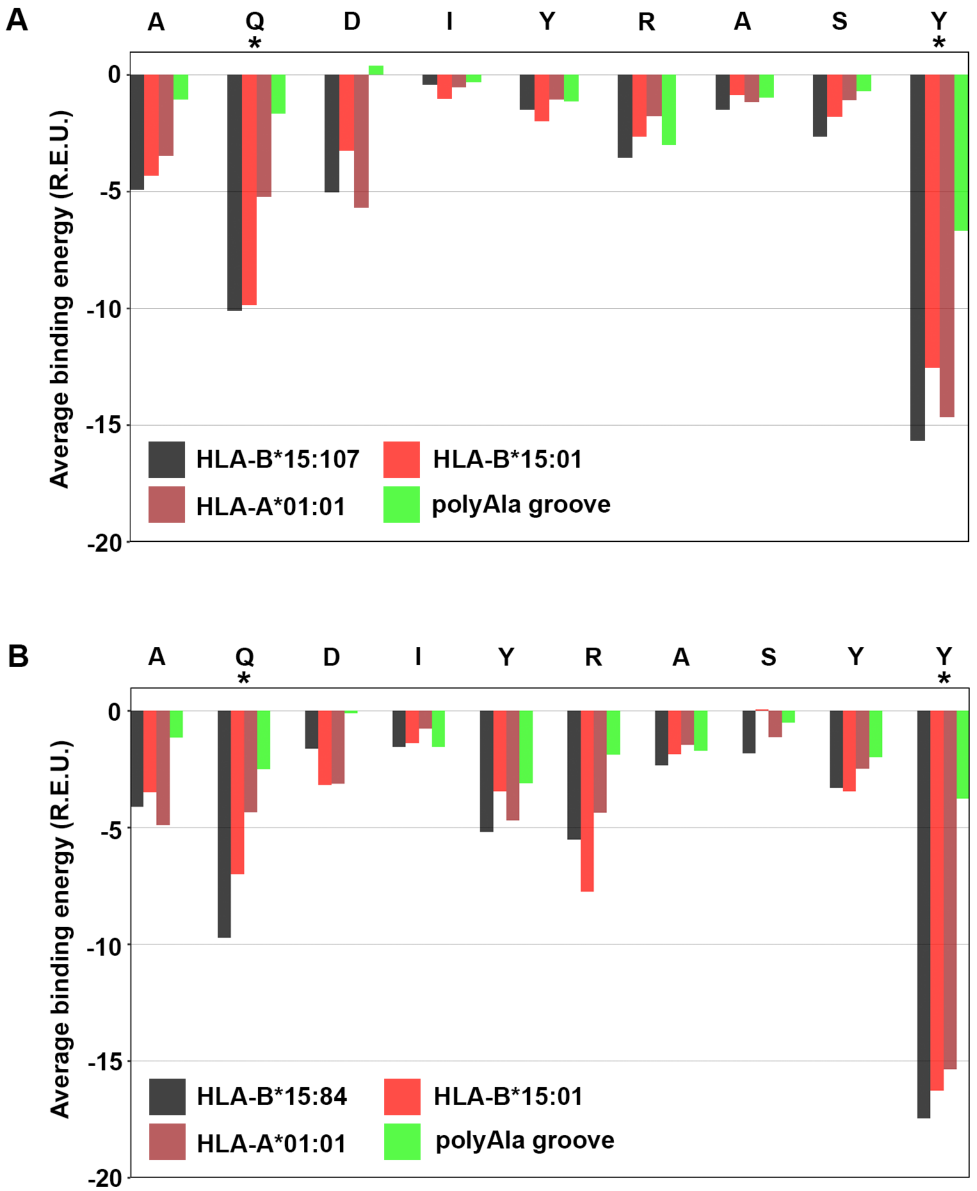

Supplementary Figure 11. Residue specific binding energy contributions for ALK nonamer AQDIYRASY and decamer AQDIYRASY peptides against different HLA grooves. *Rosetta* calculated average binding energies contributed by ALK **(A)** nonamer and **(B)** decamer residues to *(i)* top binder HLA allele, HLA-B*15:107 or HLA-B*15:84 (black), *(ii)* HLA-B*15:01 (red), *(iii)* HLA-A*01:01 (brown), and *(iv)* control HLA allele (polyAla groove, green) where all the residues in the binding groove were replaced with an alanine**.** Y-axis shows R.E.U.: *Rosetta* Energy Units. X-axis represents each peptide residue. Anchor residues are indicated by an asterisk.

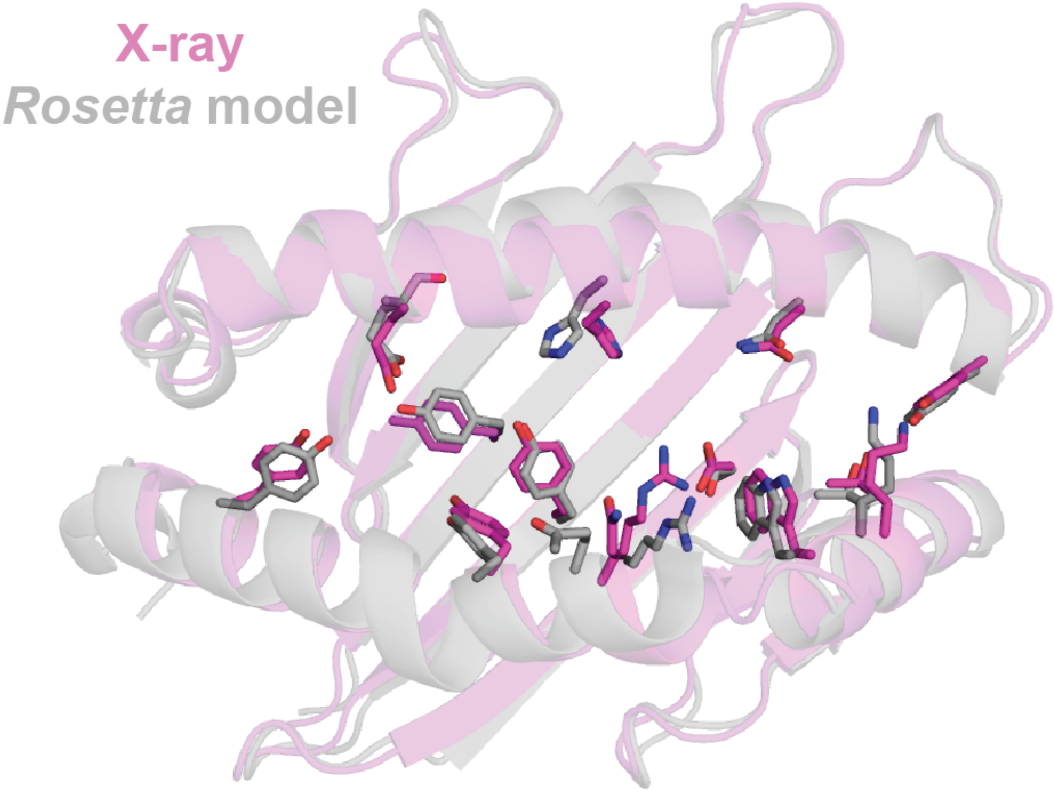

Supplementary Figure 12. Overlay of the *Rosetta* modeled and X-ray determined HLA-A*01:01 groove when bound to decamer peptide. An overlay of the *Rosetta* model (gray) and X-ray structure (pink, PDB ID 6AT9) is shown for HLA-A*01:01 when bound to AQDIYRASYY (not shown). Side-chains are represented as sticks for representative residues in contact with the peptide (see Figure 5). Structures were aligned in PyMol using backbone heavy atoms (Cα , CO N and O). The backbone and all-atom RMSD values are provided in Supplementary Table 5.

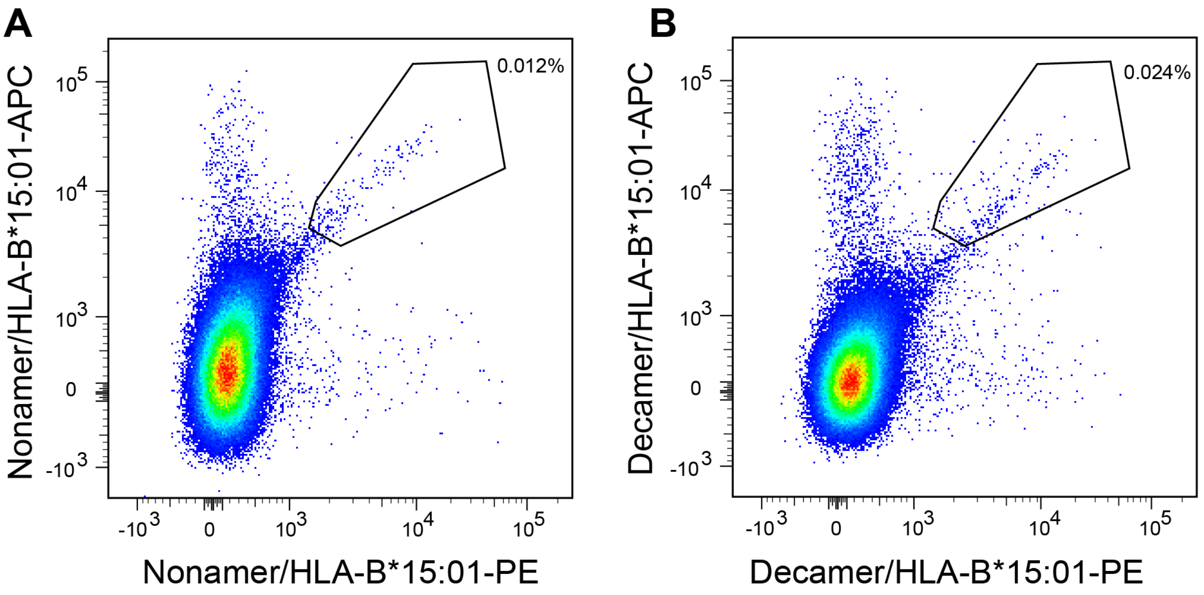

C

Donor	SARS-APC, SARS-PE	Nonamer-APC, Nonamer-PE	Decamer-APC, Decamer-PE	Decamer-APC, Nonamer-PE
#1	0.014%	0.012%	0.024%	0.000%
#2	0.007%	0.017%	0.028%	0.000%

Supplementary Figure 13. The two *ALK*-derived neoepitopes are recognized by CD8+ T cells. PMBCs stained against **(A)** nonamer/HLA-B*15:01-APC and nonamer/HLA-B*15:01-PE or **(B)** decamer/HLA-B*15:01-APC and decamer/HLA-B*15:01-PE tetramers for donor #1. The x and y-axis represent fluorescence intensity. **(C)** Summary of tetramer staining results (percentage of CD8+ T cells recognized) for independent double staining experiments against two individual HLA matched donors using *i)* SARS/HLA-B*15:01-APC and SARS/HLA-B*15:01-PE, *ii)* HLA-nonamer/B*15:01-APC and nonamer/HLA-B*15:01-PE, *iii)* decamer/HLA-B*15:01-APC and decamer/HLA-B*15:01-PE, and *iv)* decamer/HLA-B*15:01-APC and nonamer/HLA-B*15:01-PE tetramers. Live cells were gated on FSC/SSC profiles. CD8+ T cells were gated on CD8+/CD4-.

Supplementary References

1. Forbes SA, Beare D, Boutselakis H, Bamford S, Bindal N, Tate J, Cole CG, Ward S, Dawson E, Ponting L, et al. COSMIC: somatic cancer genetics at high-resolution. *Nucleic Acids Res* (2017) **45**:D777–D783. doi:10.1093/nar/gkw1121

2. Lovell SC, Davis IW, Arendall WB, de Bakker PIW, Word JM, Prisant MG, Richardson JS, Richardson DC. Structure validation by Calpha geometry: phi,psi and Cbeta deviation. *Proteins* (2003) **50**:437–450. doi:10.1002/prot.10286

3. Sievers F, Wilm A, Dineen D, Gibson TJ, Karplus K, Li W, Lopez R, McWilliam H, Remmert M, Söding J, et al. Fast, scalable generation of high-quality protein multiple sequence alignments using Clustal Omega. *Mol Syst Biol* (2011) **7**:539. doi:10.1038/msb.2011.75

4. Robert X, Gouet P. Deciphering key features in protein structures with the new ENDscript server. *Nucleic Acids Res* (2014) **42**:W320–W324. doi:10.1093/nar/gku316

5. Thomsen MCF, Nielsen M. Seq2Logo: a method for construction and visualization of amino acid binding motifs and sequence profiles including sequence weighting, pseudo counts and two-sided representation of amino acid enrichment and depletion. *Nucleic Acids Res* (2012) **40**:W281–W287. doi:10.1093/nar/gks469

6. Maccari G, Robinson J, Ballingall K, Guethlein LA, Grimholt U, Kaufman J, Ho C-S, de Groot NG, Flicek P, Bontrop RE, et al. IPD-MHC 2.0: an improved inter-species database for the study of the major histocompatibility complex. *Nucleic Acids Res* (2017) **45**:D860–D864. doi:10.1093/nar/gkw1050

7. Henikoff S, Henikoff JG. Amino acid substitution matrices from protein blocks. *Proc Natl Acad Sci U S A* (1992) **89**:10915–10919.

8. Vita R, Overton JA, Greenbaum JA, Ponomarenko J, Clark JD, Cantrell JR, Wheeler DK, Gabbard JL, Hix D, Sette A, et al. The immune epitope database (IEDB) 3.0. *Nucleic Acids Res* (2015) **43**:D405-412. doi:10.1093/nar/gku938

9. Sidney J, Assarsson E, Moore C, Ngo S, Pinilla C, Sette A, Peters B. Quantitative peptide binding motifs for 19 human and mouse MHC class I molecules derived using positional scanning combinatorial peptide libraries. *Immunome Res* (2008) **4**:2. doi:10.1186/1745-7580-4-2

10. Hoof I, Peters B, Sidney J, Pedersen LE, Sette A, Lund O, Buus S, Nielsen M. NetMHCpan, a method for MHC class I binding prediction beyond humans. *Immunogenetics* (2009) **61**:1–13. doi:10.1007/s00251-008-0341-z
